# Targeting the Undruggable: Deep Learning-Driven Design of Peptide Therapeutics in Cancer

**DOI:** 10.3390/ph19070998

**Published:** 2026-06-27

**Authors:** Ha Thi Ngoc Nguyen, Bao Hong Ngoc Le, Nhung Thi Hong Van, Trinh Thi Tuyet Tran, Minh Tuan Nguyen

**Affiliations:** 1 Faculty of Pharmacy, Lac Hong University, Dong Nai 810000, Vietnam; 2 College of Pharmacy, Dongguk University, Seoul 04620, Republic of Korea; 3 Department of Physiology, College of Medicine, Seoul National University, Seoul 03080, Republic of Korea; 4 Vinmec-VinUni Institute of Immunology, Vin University, Hanoi 100000, Vietnam

**Keywords:** undruggable, KRAS, p53, c-MYC, peptide therapeutics, deep learning, generative models, de novo design, protein–protein interaction

## Abstract

The majority of disease-associated proteins are considered “undruggable” due to the absence of well-defined binding pockets, the presence of extended interaction surfaces, and intrinsic structural disorder, which collectively limit the effectiveness of conventional small molecules and biologics. Representative examples include KRAS, p53, and c-MYC. Peptide therapeutics, particularly macrocyclic peptides, occupy a unique chemical space capable of targeting such recalcitrant protein–protein interactions (PPIs) where small molecules often fail. However, traditional peptide discovery, which relies heavily on high-throughput screening, is labor-intensive and frequently yields candidates with suboptimal pharmacological properties. The integration of artificial intelligence has begun to transform peptide discovery from a largely empirical process into a rational and design-driven paradigm. Modern deep learning approaches, including diffusion-based generative models, enable the de novo design of peptide binders with high affinity and structural precision, even for disordered or previously intractable targets. In this perspective, we highlight key structural and biological challenges associated with undruggable proteins and consider how peptide-based modalities are beginning to overcome these longstanding barriers. We further explore how advances in artificial intelligence and computational modeling may reshape the rational design of next-generation peptide therapeutics and propose an integrated experimental–computational framework to facilitate the development of clinically actionable candidates.

## 1. Introduction

**Figure 1:**
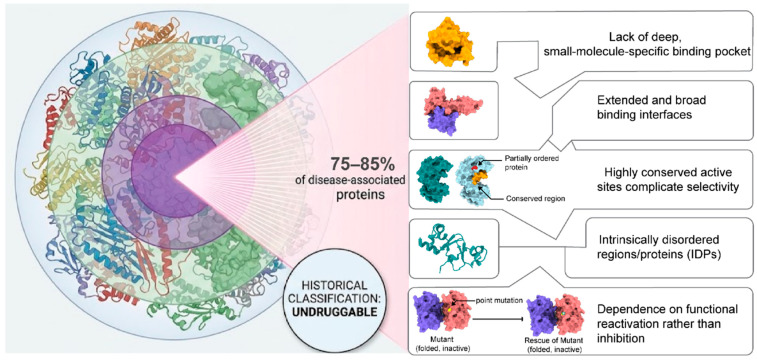
Defining features of undruggable targets. Approximately 75–85% of disease-associated human proteins are considered undruggable and share common structural and biological features.

In the context of drug discovery, the term “undruggable” refers to therapeutically relevant targets, primarily proteins, that play critical roles in disease initiation and progression but remain resistant to modulation by conventional small molecules or standard biologics. These targets typically lack the structural features required for effective ligand binding and functional modulation. Historically, druggable proteins are characterized by the presence of well-defined, deep, and narrow hydrophobic pockets that accommodate small molecules in a lock-and-key manner [1,2]. However, approximately 75% to 85% of disease-associated human proteins do not exhibit such features and are therefore classified as undruggable (Figure 1). Several structural and biological characteristics contribute to this limitation, including large and relatively flat binding interfaces, typically spanning 1500 to 3000 Å^2^, which hinder high-affinity interactions with small molecules. The presence of intrinsically disordered regions further limits the applicability of structure-based design strategies. In addition, highly conserved active sites complicate the development of selective inhibitors and increase the risk of off-target toxicity. In certain cases, these targets require functional activation rather than inhibition, a challenge that is not readily addressed by traditional drug modalities. Notably, key oncoproteins and tumor suppressors such as KRAS, p53, and c-MYC exemplify this class of historically intractable targets [3].

**Figure 2:**
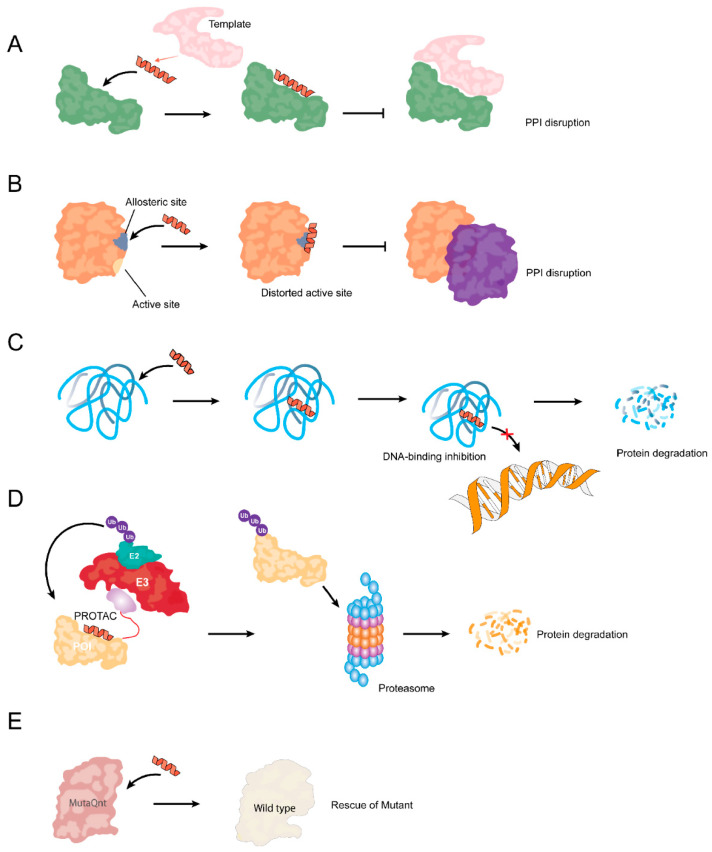
Peptide-based strategies to overcome undruggable targets. (**A**) Peptides designed based on protein–protein interaction (PPI) interfaces mimic native binding domains, enabling them to engage large, shallow binding surfaces and disrupt PPIs. (**B**) Peptides targeting allosteric sites induce conformational changes in the target protein, thereby modulating its activity with improved selectivity and disrupting PPIs. (**C**) For intrinsically disordered proteins (IDPs), peptides or miniproteins bind to disordered regions, preventing interactions with DNA and promoting degradation due to the lack of stable functional structure. (**D**) Peptide-based PROTACs recruit the ubiquitin–proteasome system to induce targeted degradation of proteins. (**E**) Peptides can restore the function of mutant proteins, such as p53, by stabilizing active conformations and promoting wild-type-like activity.

Peptides represent a promising strategy for targeting undruggable proteins, as they possess properties that bridge the gap between conventional small molecules and larger biologics such as antibodies. In particular, peptides retain key advantages of biologics, including the ability to engage broad, shallow, and highly specific interaction surfaces that are typically difficult to target with small molecules (Figure 2). At the same time, their smaller size relative to antibodies can facilitate improved cellular permeability while potentially reducing production and storage complexity [4]. Recent studies have demonstrated that peptides, particularly macrocyclic peptides, can achieve strong binding affinity toward target proteins in the nano- to micromolar range [5]. Several peptide therapeutics have progressed into clinical trials. Notably, LUNA18, a KRAS inhibitor, has demonstrated potent activity together with favorable oral bioavailability [6]. In addition, peptide-based therapeutics generally display favorable safety profiles, as they are often associated with low toxicity and low immunogenicity. Their degradation products are typically non-toxic amino acid fragments, and they are less likely to induce severe immune-related adverse effects compared with antibody-based therapies [7].

Beyond these intrinsic advantages, recent advances in artificial intelligence (AI) and deep learning have driven a shift from traditional trial-and-error methodologies toward more systematic and predictive peptide design strategies. Machine learning and generative modeling approaches now enable data-driven generation of peptide sequences, prediction of structural and binding properties, and iterative optimization of candidate performance. In parallel, the convergence of generative AI, molecular simulation, and experimental validation is accelerating the discovery of functional peptides against challenging targets, including protein–protein interactions and intrinsically disordered proteins. As these technologies continue to mature, AI-assisted design is poised to become an integral component of next-generation peptide therapeutic development [3,8,9,10].

This perspective explores the emerging role of advanced deep learning architectures in the de novo design of peptides targeting historically intractable biological systems. We begin by considering the unique challenges associated with traditionally undruggable targets and highlighting recent advances in peptide-based therapeutics for these systems. We then outline the evolution of computational peptide design strategies, spanning early sequence-based models to emerging generative AI frameworks. Building on these developments, we further propose an integrated computational workflow for the de novo design and optimization of functional peptides. Finally, we discuss current limitations, outstanding challenges, and future directions that may shape the next generation of AI-driven peptide therapeutics.

## 2. Peptide Therapeutics: A Promising Modality for Undruggable Targets

### 2.1. Small Gtpase Kras: Targeting the Undruggable Master Regulator

#### 2.1.1. Structural Insights and Biological Functions of Kras

KRAS (Kirsten rat sarcoma viral oncogene homolog) is a proto-oncogene encoding a small GTPase that operates as a binary molecular switch controlling key cellular processes. Oncogenic KRAS mutations are present in ~30% of human cancers, particularly pancreatic, colorectal and lung malignancies, and most frequently affect codons 12, 13 and 61. Despite its central role in oncogenesis, KRAS was long considered undruggable owing to the absence of deep, druggable binding pockets and its exceptionally high affinity for GTP, which limits effective competition by small molecules [11,12,13].

KRAS is a member of the RAS family, which also includes HRAS and NRAS. It expresses two splice variants, KRAS4A and KRAS4B, with the latter being the predominant isoform in human cells. Structurally, KRAS is a 21 kDa protein comprising a catalytic G-domain (residues 1–166) and a C-terminal hypervariable region (HVR; residues 167–189). The G-domain comprises the P-loop and the conformationally dynamic Switch I and Switch II regions that mediate nucleotide-dependent structural transitions. Together, these elements constitute the effector lobe, the principal binding interface for effector proteins and RAS regulators. It also includes an allosteric lobe implicated in regulatory and membrane-associated functions. The C-terminal hypervariable region contains a CAAX motif that undergoes post-translational modifications, enabling membrane anchoring required for KRAS signaling [14].

Functionally, KRAS cycles between an inactive GDP-bound state and an active GTP-bound state. This process is regulated by guanine nucleotide exchange factors (GEFs; such as SOS1/2), which promote GDP release and GTP loading, and GTPase-activating proteins (GAPs; such as NF1), which accelerate GTP hydrolysis. In the GTP-bound state, stabilization of Switch I and Switch II by the γ-phosphate generates a high-affinity interface for effectors such as RAF, PI3Kα and RALGDS, thereby promoting proliferation and survival. Signal termination depends on GTP hydrolysis, which returns KRAS to its inactive state [15,16,17].

Oncogenic KRAS mutations disrupt this regulatory cycle by impairing GTP hydrolysis and conferring resistance to GAP-mediated inactivation, resulting in constitutive signaling. Mutations at codons 12 and 13 (including G12D, G12V, G12C and G13D) introduce steric constraints that impair GAP binding, whereas substitutions at codon 61 (e.g., Q61H) directly compromise the catalytic machinery of GTP hydrolysis. Mutation frequencies are context-dependent, 80–90% in pancreatic cancer (predominantly G12D), 30–50% in colorectal cancer, and 15–35% in lung adenocarcinoma, where G12C is enriched in smokers and G12D in never-smokers [12,13,18,19].

A major breakthrough occurred with the identification of an inducible allosteric pocket beneath the Switch II region (the Switch II pocket, S-IIP), enabling the development of mutant-selective inhibitors targeting KRAS^G12C^ such as MRTX849 and AMG 510. Covalent inhibitor such MRTX849 exploit the cysteine substitution at codon 12, forming irreversible bonds with its thiol group and achieving high allele selectivity while sparing wild-type KRAS. Notably, KRAS^G12C^ retains partial GTPase activity, allowing nucleotide cycling and enabling inhibitors to preferentially bind and trap the inactive GDP-bound state [19,20,21].

By contrast, other prevalent mutants, including KRAS^G12D^ and KRAS^G12V^, remain considerably more challenging to target. These variants lack a reactive cysteine residue, limiting covalent strategies, and their substituted side chains (aspartate or valine) are chemically less amenable to small-molecule engagement. Moreover, their markedly impaired GTPase activity results in a predominance of the active GTP-bound state, reducing accessibility to the inactive conformation targeted by current inhibitors. Together, these structural and biochemical constraints underpin the persistent difficulty in directly targeting non-G12C KRAS mutants and highlight the need for alternative therapeutic strategies [18,21]. Because these mutants present relatively flat and featureless protein surfaces rather than deep druggable pockets, overcoming these limitations may require therapeutic modalities capable of engaging larger interaction interfaces.

#### 2.1.2. Potential Kras Peptide Inhibitors

To overcome the structural constraints of non-G12C mutants, therapeutic development has increasingly shifted toward macromolecular modalities. In this context, peptides have emerged as promising modulators of KRAS signaling due to their intermediate size and structural versatility. Compared with small molecules, peptides provide a larger interaction surface, enabling them to engage the relatively flat and hydrophobic interfaces that characterize many protein–protein interactions. This feature allows peptides to span extended contact areas and disrupt critical interactions between KRAS and its regulatory or effector proteins, including SOS1, RAF and PI3K [22,23].

In addition, peptides can achieve exceptionally high binding affinities, often in the nanomolar or sub-nanomolar range, and can exhibit remarkable selectivity towards oncogenic KRAS variants, including G12D, G12V and G12C, relative to wild-type KRAS [5]. Such selectivity offers the potential to preferentially inhibit mutant-driven signaling in cancer cells while minimizing disruption of normal KRAS function in healthy tissues.

*Disruption of KRAS protein–protein interactions through helix-mimetic stapled peptides*

One of the earliest mechanistically defined strategies involves mimicking the α-helical interface of SOS1 that engages KRAS. The stapled peptide SAH-SOS1_A_, derived from residues 929–944 of SOS1, adopts a pre-organized helical conformation that enables high-affinity binding to KRAS [24]. Subsequent minimization of this scaffold produced Stapled Peptide 3, which retains the helical binding mode and introduces hydrophobic contacts with residues surrounding Cys12. This additional interaction enhances binding affinity and stabilizes the peptide–KRAS complex. Functionally, this results in effective blockade of the KRAS-RAF-MEK-ERK cascade, inducing G2/M arrest and apoptosis in KRAS^G12C^-mutant cells [25].

A related structure-guided strategy targeting the KRAS-RAF interface further illustrates this principle. Peptides derived from CRAF initially exhibited weak affinity, but hydrocarbon stapling markedly increased helicity and binding potency. To address the limited membrane permeability typically associated with hydrophilic peptides, a stearic acid moiety was introduced at the N-terminus to enhance membrane association and cellular uptake. This lipidation strategy led to the development of the optimized derivatives Sraf-2-1 and Sraf-7-1, which suppressed p-AKT signaling, induced apoptosis, and inhibited cancer cell migration [26].

*Targeting the switch II pocket with cyclic peptides*

In addition to PPI disruption, peptides have enabled access to allosteric sites on KRAS. The discovery of KRpep-2d represents a key milestone, providing a high-affinity structural template for subsequent optimization aimed at improving metabolic stability, cellular permeability, and in vivo efficacy. KRpep-2d was identified using T7 phage display against recombinant KRAS^G12D^ in its inactive GDP-bound state. The peptide, stabilized by a disulfide bond between Cys5 and Cys15, exhibited nanomolar potency and strong selectivity for KRAS^G12D^ over wild-type and other mutants. Structural analysis revealed that KRpep-2d binds to an allosteric pocket near the Switch II region, thereby sterically blocking interactions between KRAS and its binding partners [27,28].

Building on this scaffold, KS-58 incorporates bicyclic constraints and non-natural residues to enhance rigidity and proteolytic stability. It acts as a potent non-covalent inhibitor with high selectivity for KRAS^G12D^, with a binding EC_50_ of approximately 22 nM, whereas other KRAS mutants exhibit binding EC_50_ values of 47–160 nM. KS-58 retains the ability to bind both GDP- and GTP-bound KRAS, suggesting recognition of a nucleotide-state-independent structural feature. By disrupting both KRAS-SOS1 and KRAS-BRAF interactions, KS-58 achieves broader pathway inhibition and demonstrates in vivo efficacy, although rapid clearance remains a limitation [23,29].

High-throughput optimization strategies further refined this approach. Using Automated Ligand Identification System (ALIS)-based screening, Peptide 7 incorporates rigid aromatic residues that enhance packing within the Switch II pocket, while replacement of the disulfide bond with a methylene bridge eliminates redox sensitivity. These modifications improve both binding affinity and intracellular stability, enabling effective suppression of ERK phosphorylation in KRAS^G12D^-mutant AsPC-1 pancreatic cancer cells, without detectable cytotoxicity or membrane disruption in RAS-independent cell lines [30].

*Targeting the active state: cyclorasin peptides as Ras–effector disruptors*

Another important application of peptides is the direct inhibition of Ras–effector interactions. The cyclorasin series exemplifies this approach, with cyclic and bicyclic peptides designed to selectively bind the active Ras-GTP state and block interactions with downstream effectors such as RAF and PI3K. Cyclorasin 9A5 contains an amphipathic motif (Arg-Arg-D-Nal-Arg-Fpa) that facilitates membrane penetration, while the remaining sequence was identified through random screening. Cyclorasin 9A5 preferentially binds the active Ras-GTP state (K_d_ = 0.44 μM), showing a 5.7-fold selectivity over the inactive Ras-GDP state [31].

Further optimization led to the bicyclic peptide B4-27, which exhibits substantially enhanced affinity and stability. Bicyclization reduces conformational flexibility, thereby increasing binding efficiency and resistance to proteolysis. Functionally, B4-27 acts as a pan-Ras inhibitor by targeting a conserved effector-binding interface across multiple isoforms and mutations. It effectively reduces the viability of Ras-mutant cancer cell lines (EC_50_ = 0.5–12 μM), whereas effects in noncancerous cells carrying wild-type Ras are observed only at higher concentrations (EC_50_ ≥ 20 μM) [5].

*Inducing druggable pockets: macrocyclic peptides and conformational remodeling*

**Figure 3:**
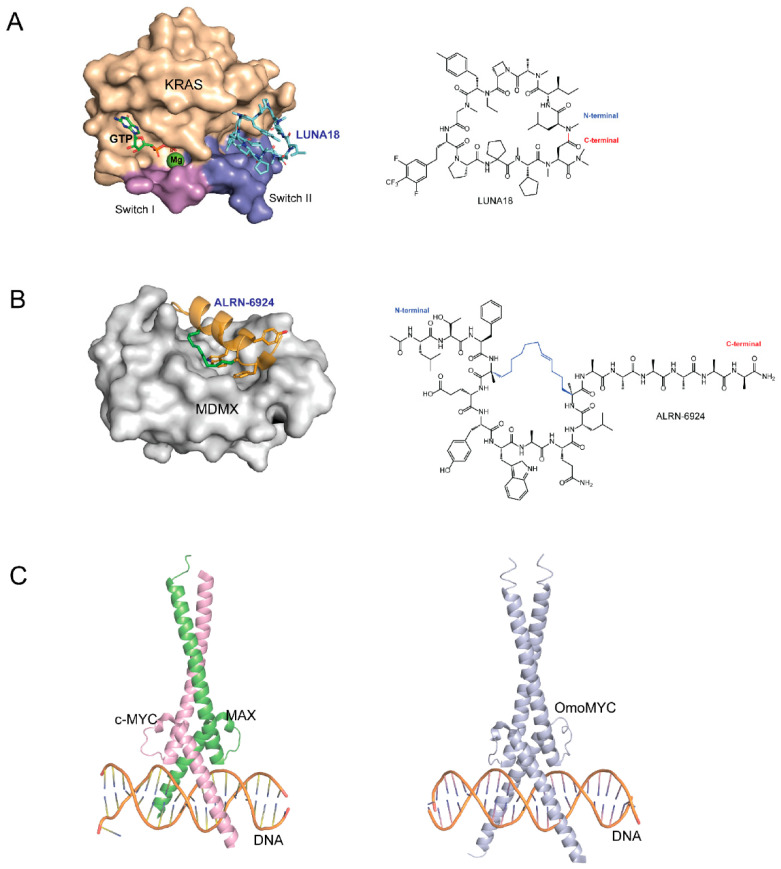
Representative peptide-based modulators of undruggable targets. (**A**) Structure of the cyclic peptide LUNA18 bound near the Switch II region of KRAS (**left**; PDB ID: 7YV1) and the chemical structure of LUNA18 (**right**). (**B**) Structure of ALRN-6924 bound to MDMX (**left**; PDB ID: 8GJS) and the chemical structure of ALRN-6924 (**right**). (**C**) Structure of the MYC-MAX heterodimer bound to DNA (**left**; PDB ID: 1NKP) and the homodimer Omomyc, a dominant-negative MYC mutant and precursor of OMO-103, bound to DNA (**right**; PDB ID: 5I50).

A key limitation of KRAS targeting is the absence of stable binding pockets. Macrocyclic peptides such as LUNA18 address this challenge by inducing new binding sites. Structural analysis revealed that its precursor binds near the Switch II region and triggers a conformational rearrangement that generates a previously unobserved cavity, termed the SII-hole (Figure 3A).

The discovery originated from a specialized mRNA display library designed to favour drug-like properties, including short peptide length (9–11 residues), extensive N-alkylation, high lipophilicity, and the absence of charged functional groups. Subsequent optimization improved rigidity, lipophilicity, and membrane permeability, ultimately yielding LUNA18. This compound exhibits strong cellular activity (IC_50_ = 0.17–2.9 nM), and oral bioavailability, and has advanced to Phase I clinical trials [6]. Despite the promising preclinical and early clinical profile of LUNA18, its development was discontinued by Chugai Pharmaceutical due to a relatively narrow therapeutic window compared with competing KRAS inhibitors. However, subsequent optimization of the LUNA18 scaffold led to the development of AUBE00, a next-generation pan-KRAS inhibitor that has entered Phase I clinical trials (NCT07030959).

Collectively, peptide-based inhibitors do not simply bind KRAS but actively reshape its interaction landscape. By stabilizing specific conformations, occluding binding interfaces, or inducing transient pockets, these molecules provide multiple routes to suppress KRAS signaling (Table 1). This mechanistic versatility distinguishes peptides from traditional small molecules and underscores their potential as a platform for targeting otherwise intractable oncogenic proteins.

### 2.2. The p53 Tumor Suppressor: Reactivating the Guardian of the Genome

#### 2.2.1. Structural and Functional Features of p53

The tumor suppressor protein p53 is a multidomain phosphoprotein comprising 393 amino acids in humans and is encoded by the *TP53* gene located on chromosome 17p13.1. Often referred to as the “guardian of the genome,” p53 plays a central role in maintaining genomic integrity [48].

Structurally, p53 is organized into five distinct functional domains. The N-terminal transactivation domain (TAD; residues 1–61) is intrinsically disordered but can adopt an amphipathic α-helix upon binding to partner proteins such as MDM2. It is subdivided into TAD1 and TAD2, both of which are essential for recruitment of the transcriptional machinery. Adjacent to this region, the proline-rich domain (PRD; residues 62–94) contains multiple PXXP motifs and contributes to p53 stability as well as its ability to induce apoptosis and growth arrest. The central DNA-binding domain (DBD; residues 95–292), also known as the core domain, mediates sequence-specific DNA recognition. This domain adopts an immunoglobulin-like β-sandwich structure with loop regions stabilized by a zinc ion, and notably, over 90% of tumor-associated mutations occur within this region. The tetramerization domain (TD; residues 325–356) facilitates the formation of a functional tetramer, which is required for effective DNA binding. Finally, the C-terminal regulatory domain (CTD; residues 357–393) is an unstructured, basic region involved in nonspecific DNA interactions, cofactor recruitment, and regulation of protein stability through multiple post-translational modifications [48,49,50,51].

Functionally, p53 acts as a central transcription factor that integrates diverse cellular stress signals to determine cell fate. In response to DNA damage, p53 induces cell cycle arrest at the G1 phase or at the G2/M phase, thereby allowing time for DNA repair [48]. When damage is irreparable, p53 promotes apoptosis through transcriptional activation of pro-apoptotic genes such as PUMA, BAX, and NOXA, as well as through transcription-independent interactions with mitochondrial Bcl-2 family proteins [48,50]. In addition, p53 directly regulates DNA repair processes by activating genes such as DDB2 and XPC [49], and can induce cellular senescence to prevent the proliferation of potentially malignant cells [50]. Beyond these canonical roles, p53 has also been implicated in the regulation of cellular metabolism, ferroptosis, autophagy, and the tumor immune microenvironment [48,49].

The activity of p53 is tightly controlled by its negative regulators MDM2 and MDMX. MDM2, an E3 ubiquitin ligase, binds to the TAD of p53, thereby masking its interaction with the transcriptional machinery and targeting it for proteasomal degradation. MDMX, a structural homolog of MDM2, further suppresses p53 transcriptional activity and enhances MDM2-mediated degradation. Under stress conditions, these inhibitory interactions are disrupted, leading to stabilization and activation of p53 [49].

Despite its critical role, p53 is frequently inactivated in cancer. More than 50% of human cancers harbor mutations or deletions in *TP53*, while in many of the remaining cases, wild-type p53 function is compromised through overexpression of its negative regulators, MDM2 and MDMX [32,50]. Consequently, p53 has become a major focus in oncology and drug discovery. However, it has historically been classified as undruggable due to several factors. First, p53 lacks well-defined hydrophobic binding pockets, instead presenting a relatively flat surface dominated by large protein–protein interaction interfaces, which are challenging for small-molecule targeting. Second, therapeutic strategies often require restoration of p53 function rather than inhibition, which is fundamentally more difficult. Third, p53 is intrinsically unstable, with a high turnover rate, and mutations frequently exacerbate this instability, leading to misfolding or aggregation [3,41,50].

Finally, targeting the p53 pathway poses significant safety challenges, as p53 is also expressed in normal tissues. Systemic activation of p53 can result in dose-limiting toxicities, including thrombocytopenia and neutropenia, due to p53-mediated cell death in normal hematopoietic cells [52].

#### 2.2.2. Peptide-Based Strategies for Targeting the p53 Pathway

Peptides have emerged as an effective approach to address the historically undruggable nature of p53. Due to their ability to mimic the large and relatively flat protein–protein interaction surfaces of p53, peptide-based molecules can modulate interactions that are typically inaccessible to conventional small molecules. As a result, peptides are employed for several key therapeutic strategies within the p53 pathway.

*Inhibition of the p53-MDM2/MDMX interaction*

Peptides constitute a highly effective class of inhibitors targeting the interaction between p53 and its primary negative regulators, MDM2 and MDMX (MDM4). In its native state, p53 binds to hydrophobic pockets on MDM2 and MDMX through an N-terminal α-helical motif. This interaction is critically dependent on three residues (Phe19, Trp23, and Leu26), collectively termed the “core triad”. Rationally designed peptide inhibitors recapitulate this structural motif, presenting these residues in an optimal spatial arrangement to occupy the MDM2/MDMX binding clefts, thereby displacing endogenous p53 and promoting its stabilization and tumor-suppressive activity [34]. To overcome the inherent limitations of conventional peptides, including proteolytic instability and poor membrane permeability, various structural modifications have been introduced.

SAH-p53-8 represents a pioneering 16-residue stapled α-helical peptide, in which an all-hydrocarbon staple is introduced across the helical interface (positioned before Asn20 and after Leu26). This modification stabilizes the α-helical conformation and enhances cellular uptake. Functionally, SAH-p53-8 acts as a dual inhibitor of both MDM2 and MDMX, addressing a key limitation of many small-molecule inhibitors that fail to effectively target MDMX, which can contribute to therapeutic resistance in tumors with high MDMX expression. The peptide exhibits potent cytotoxicity against cancer cells overexpressing MDM2, MDMX, or both. Structurally, the hydrocarbon staple not only reinforces helicity but also directly contributes to binding by forming hydrophobic contacts with residues at the rim of the MDM2/MDMX pocket (Leu54, Phe55, Gly58, and Met62), accounting for approximately 10% of the total interaction surface. However, its activity is significantly reduced in the presence of serum [32]. Nevertheless, SAH-p53-8 provided a foundational scaffold for the development of more advanced stapled peptides. Subsequent iterative optimization yielded ATSP-7041 and, ultimately, ALRN-6924, the first stapled peptide to enter clinical evaluation.

ATSP-7041 provided the first demonstration that stapled peptides can effectively activate p53 in vivo. A key advancement in its design was the incorporation of Tyr22, identified through phage display screening. This residue enhances binding affinity through multiple interactions, including hydrophobic and electrostatic contacts, π-cation interactions, and water-mediated hydrogen bonding with residues such as Lys90 and His69 in MDMX [34].

ALRN-6924 is a 17-amino-acid stapled α-helical peptide developed through iterative structural optimization to overcome the pharmacological limitations of linear peptides. It contains a penta-L-alanyl-D-alaninamide C-terminal extension that confers a “chameleonic” property, enabling the molecule to remain soluble in aqueous environments while adopting a helical conformation under hydrophobic conditions, thereby facilitating membrane permeation (Figure 3B). Functionally, ALRN-6924 acts as a prodrug: upon cellular entry, it undergoes intracellular proteolysis to generate a 14-residue metabolite, ALRN-8714, with approximately 10-fold higher binding affinity toward both MDM2 and MDMX than the parent peptide. The compound exhibits dose-dependent biological effects, enabling both anticancer activity at higher doses and chemoprotective (cyclotherapy) effects at lower doses. Clinically, ALRN-6924 has been evaluated in more than 200 patients across Phase I and II trials in multiple malignancies, including solid tumors, lymphomas, acute myeloid leukemia, and myelodysplastic syndrome [33]. In a Phase I clinical trial (NCT02264613), the treatment was generally well tolerated, with nausea (73.2%), fatigue (60.6%), and vomiting (46.5%) being the most frequently reported treatment-related adverse events. Notably, Grade 3/4 thrombocytopenia, a common dose-limiting toxicity associated with small-molecule MDM2 inhibitors, was rarely observed. Among 41 efficacy-evaluable patients, the treatment achieved a disease control rate of 59%. Collectively, these findings highlight the therapeutic potential of stapled peptides as an alternative strategy for targeting the p53-MDM2/MDMX signaling axis [53].

In addition to classical stapled peptides, alternative designs such as bicyclic stapled peptides, di-alkyne stapled peptides, sulfono-γ-AApeptides, and D-peptides have been explored to further enhance stability, binding affinity, and cellular permeability, thereby improving the therapeutic potential of peptide-based PPI inhibitors [35,36,37,38].

PROTACs (proteolysis-targeting chimeras) represent a transformative approach in drug discovery, shifting the paradigm from conventional inhibition to targeted protein degradation. Building on this concept, stapled peptide-based PROTACs have emerged as a promising strategy, exemplified by SPMI-HIF2-1. Rather than simply inhibiting the p53-MDM2/MDMX interaction, these bifunctional molecules promote the degradation of MDM2 and MDMX via the ubiquitin–proteasome system, resulting in sustained activation of the p53 pathway. SPMI-HIF2-1 comprises three components: (i) a targeting moiety derived from a hydrocarbon-stapled PMI-N8A peptide (TSFAEYWALLSP), which binds both MDM2 and MDMX; (ii) a recruiting moiety consisting of a hexapeptide motif (LA-Hyp-Y-Hle-P) that engages the VHL E3 ubiquitin ligase; and (iii) a polyethylene glycol linker that connects the two components to facilitate ternary complex formation. This molecule selectively induces cytotoxicity in cancer cells harboring wild-type p53 (e.g., HCT116 p53^+/+^), while showing minimal toxicity in p53-deficient cells [39].

*Peptide-mediated structural rescue of mutant p53*

Mutant p53 is commonly inactivated through structural destabilization and aggregation. Beyond inhibition of regulatory interactions, peptides have also been developed to restore the function of mutant p53.

Many missense mutations in the DNA-binding domain lead to partial unfolding of the protein, exposing an aggregation-prone segment (residues 252–258, LTIITLE) that promotes self-association into inactive aggregates. ReACp53 is a peptide designed to inhibit this aggregation process through a modified sequence (LTRITLE), which introduces steric hindrance and thereby prevents further aggregation. By masking exposed hydrophobic regions, ReACp53 shifts the equilibrium toward a soluble, properly folded conformation. This results in redistribution of p53 from cytosolic aggregates to the nucleus, restoring its transcriptional activity. The peptide has demonstrated efficacy in patient-derived organoids of high-grade serous ovarian carcinoma and in mutant p53-expressing prostate cancer models, significantly reducing tumor burden in vivo [40].

A complementary approach involves p53 conformation-activating peptides (pCAPs), identified through phage display screening of large peptide libraries. These peptides stabilize the small fraction of transiently folded, functional p53 molecules rather than directly refolding misfolded proteins. By binding to these conformers, pCAPs prevent unfolding and progressively shift the equilibrium toward an active state. This mechanism results in increased expression of canonical p53 target genes, induction of apoptosis in mutant p53-expressing cells, and significant tumor regression in xenograft models of breast, ovarian, and colon cancers [41].

Although both ReACp53 and pCAPs aim to restore the activity of mutant p53 proteins in cancer cells, they appear to operate through distinct mechanisms. ReACp53 primarily targets aggregation-prone mutant p53 variants, whereas pCAPs are designed to modulate misfolded p53 conformations. Notably, pCAPs do not directly refold mutant proteins but instead shift the conformational equilibrium toward an active state by preferentially binding to the rare, properly folded conformation. Both approaches have shown encouraging antitumor effects in breast cancer models, with reported tumor reductions of approximately 80–90% [40,41].

Peptide-based strategies provide a versatile platform for modulating the p53 pathway, enabling inhibition of p53-MDM2/MDMX interactions, targeted protein degradation, and restoration of mutant p53 function (Table 1). Advances in understanding p53 structural dynamics are expected to facilitate the development of more selective and clinically effective peptide therapeutics. Collectively, these approaches position peptide-based modalities as a promising avenue for reactivating p53 and improving outcomes in difficult-to-treat cancers.

### 2.3. The C-Myc Transcription Factor: Disrupting the Hub of Oncogenic Transcription

#### 2.3.1. Molecular Architecture and Dimerization Mechanisms of C-Myc

The MYC family of transcription factors, comprising c-MYC, N-MYC, and L-MYC, functions as a central regulator of global gene expression. Among these, c-MYC is the most ubiquitously expressed and frequently deregulated member, with an estimated involvement in up to 70% of human cancers [54]. As a master regulator, MYC controls approximately 15–33% of the human genome, underscoring its pivotal role in cellular homeostasis [54,55]. While c-MYC is broadly expressed in proliferating cells, N-MYC and L-MYC exhibit more tissue-specific functions, particularly in neural and lung development, respectively [56]. In normal physiological conditions, MYC expression is tightly regulated, with a notably short protein half-life of 15–30 min, allowing rapid responsiveness to growth [42,57].

Structurally, MYC is a 62 kDa protein composed of 439 amino acids and organized into three principal functional regions. The N-terminal domain contains the transcriptional transactivation domain (TAD) and conserved MYC boxes (MB0, MBI, MBII), which are essential for the recruitment of transcriptional cofactors and include critical phosphorylation sites such as Serine 62, associated with protein stabilization, and Threonine 58, which promotes degradation. The central region comprises a PEST domain linked to rapid protein turnover, additional MYC boxes (MBIII and MBIV), and a nuclear localization sequence (NLS) required for nuclear import. The C-terminal region contains the basic helix–loop–helix leucine zipper (bHLHZip) domain, which mediates protein dimerization and sequence-specific DNA binding [42,56].

Functionally, MYC is inactive as a monomer and requires heterodimerization with its obligate partner MAX to exert its transcriptional activity. This interaction is mediated through the bHLHZip domains, forming a stable complex that binds DNA. The MYC-MAX heterodimer recognizes specific enhancer-box (E-box) sequences, typically the palindromic motif 5′-CACGTG-3′, enabling regulation of downstream target gene [42,58,59]. Through this mechanism, MYC acts as a global amplifier of transcription, coordinating multiple cellular programs. It promotes cell cycle progression by activating cyclins and CDK4 while repressing cell cycle inhibitors such as p21 and p27. In parallel, MYC drives metabolic reprogramming, enhancing glycolysis, glutaminolysis, and lipid biosynthesis to support rapid proliferation, a hallmark consistent with the Warburg effect. Additionally, it stimulates ribosome and mitochondrial biogenesis, thereby increasing cellular biomass. Beyond intrinsic cellular functions, MYC also contributes to immune evasion by upregulating molecules such as CD47 and PD-L1. Notably, MYC exhibits a dual functional role: although it promotes proliferation, it can also sensitize cells to apoptosis under unfavorable conditions, serving as a safeguard against uncontrolled growth [54,55,57].

Despite its central role in oncogenesis, MYC has long been considered a prototypical undruggable target. As an intrinsically disordered protein, MYC lacks a stable three-dimensional structure in its monomeric form and instead exists as a dynamic ensemble of conformations. This structural plasticity results in the absence of well-defined binding pockets and the presence of relatively smooth interaction surfaces, limiting opportunities for high-affinity small-molecule binding. Furthermore, MYC is capable of undergoing liquid–liquid phase separation to form membrane-less condensates within the nucleus, which may further restrict the accessibility of therapeutic agents. Its predominant nuclear localization introduces an additional barrier to drug delivery. Compounding these challenges, both MYC protein and its mRNA exhibit extremely short half-lives, allowing rapid resynthesis that can outpace pharmacological inhibition and complicate the maintenance of effective intracellular drug concentrations. Biologically, MYC is indispensable for normal cellular growth, proliferation, and tissue regeneration, resulting in a narrow therapeutic window. Moreover, unlike many oncogenes such as KRAS or BRAF, MYC is rarely mutated in human cancers; instead, its oncogenic activity primarily arises from dysregulated expression of the wild-type protein, thereby limiting the feasibility of mutant-selective therapeutic strategies [42,45,58].

#### 2.3.2. Peptide and Miniprotein Approaches for Targeting C-Myc

Given that c-MYC is the most ubiquitously expressed and frequently deregulated member in human cancers, it has become a primary target, with peptides and miniproteins being extensively investigated to modulate its activity. Current research efforts have focused on developing peptide-based strategies that target c-MYC through distinct mechanisms, primarily by disrupting protein–protein interactions or by directly blocking DNA binding.

*Disruption of MYC-MAX interaction*

One major strategy involves inhibition of the interaction between c-MYC and its obligate partner MAX, a prerequisite for transcriptional activity. Several peptides have been designed to disrupt this interaction. A notable example is OMO-103, a 91-amino acid therapeutic miniprotein derived from Omomyc, a dominant-negative mutant of MYC (Figure 3C). Miniproteins are small polypeptide chains (1–10 kDa) that possess highly stable three-dimensional structures, stabilized by dense disulfide networks or tight hydrophobic cores [60]. The targeting capability of OMO-103 is based on four specific amino acid substitutions E410T, E417I, R423Q, and R424N in the leucine zipper domain, which eliminate electrostatic repulsion and enable homodimer formation. As a result, OMO-103 can homodimerize, heterodimerize with MAX, and heterodimerize with MYC. Mechanistically, OMO-103 binds directly to MYC to form inactive OMO/MYC heterodimers that are unable to bind DNA. At the same time, OMO-103 forms homodimers and heterodimers with MAX that retain high-affinity binding to E-box sequences but lack transactivation domains, thereby acting as trans-repressive complexes that occupy DNA and prevent binding of functional MYC/MAX complexes. Disruption of the MYC/MAX interaction also results in the accumulation of unstable MYC monomers, which are subsequently targeted for ubiquitination and proteasomal degradation [42,54]. Importantly, OMO-103 exhibits intrinsic cell-penetrating properties, as its basic region functions as a protein transduction domain, enabling uptake via endocytosis and macropinocytosis and efficient nuclear localization. This capability has been confirmed in a Phase I clinical trial, demonstrating successful target engagement in human patients [42,61].

IDP-121 is an engineered stapled peptide developed to directly target and inhibit the c-MYC protein. The structural details of IDP-121 remain undisclosed. However, biochemical studies have demonstrated that the peptide binds selectively to c-MYC, with a K_d_ of approximately 400 nM. This affinity is reportedly ten-fold higher than that of the native c-MYC/MAX interaction, enabling effective displacement of MAX from the complex. Disruption of the MYC/MAX heterodimer leaves c-MYC in a destabilized monomeric state, ultimately promoting its proteasomal degradation. In preclinical studies, IDP-121 exhibited potent anti-tumor activity against multiple myeloma, significantly reducing cell viability and inducing apoptosis. Furthermore, the peptide enhanced the therapeutic efficacy of standard multiple myeloma treatments, including bortezomib and dexamethasone. IDP-121 has subsequently advanced into clinical development and is currently being evaluated in a Phase I/II clinical trial (NCT05908409) for patients with MYC-driven hematological malignancies [43,44,56].

Beyond the peptide and mini-protein candidates currently undergoing clinical evaluation, additional efforts have also been directed toward targeting c-MYC. One notable example is NT-B2R, a bicyclic peptide generated from a stereodiversified library and designed to provide the rigidity and hydrophobicity required to bind the smooth and intrinsically disordered surface of c-Myc. NT-B2R was synthesized using a tandem ring-opening metathesis and ring-closing metathesis (ROM-RCM) strategy, enabling one-step cyclization of a linear peptide. Specifically, NT-B2R corresponds to the endo-R isomer, which demonstrated substantially higher binding affinity toward MYC compared with the corresponding endo-S isomer (NT-B2S). Mechanistically, the peptide binds specifically to the E363-R378 epitope of c-Myc, a region critical for MYC-MAX heterodimerization and subsequent DNA binding, and exhibits high-nanomolar binding affinity. Importantly, NT-B2R functions primarily as a transcriptional inhibitor rather than a degrader, leading to transcriptional suppression without altering MYC protein expression, while also inhibiting cancer cell growth [45].

*Direct Blockade of E-Box DNA Binding*

An alternative strategy focuses on directly blocking MYC binding to DNA. Instead of targeting protein–protein interactions, certain engineered peptides and miniproteins are designed to occupy E-box sequences where c-MYC normally binds. ME47, also known as MAX-E47, is a representative miniprotein of 66 amino acids, substantially smaller than the native MAX protein. It is constructed by combining the DNA-binding basic region of MAX with the helix–loop–helix domain of the transcription factor E47. ME47 forms dimers and binds E-box sequences such as CACGTG with high affinity, comparable to native MAX. By occupying these sites on chromatin, it prevents the oncogenic c-MYC/MAX complex from accessing its target genes. Notably, unlike Omomyc-derived constructs, ME47 does not directly interact with c-MYC or MAX, providing a selective mechanism that specifically blocks MYC binding to DNA [46,62].

In a similar manner, Max* is a miniprotein derived from the full bHLHZip domain of MAX and consists of 83 amino acid residues. It contains a strongly positively charged nuclear localization sequence KRAHHNALERKRR within its basic region, which enables intrinsic cell penetration through clathrin- and dynamin-dependent endocytosis, followed by accumulation in the nucleus. Max* forms homodimers that compete with c-MYC/MAX complexes for binding at E-box sites, thereby inhibiting MYC-driven transcriptional activation [47].

Collectively, these approaches illustrate how peptides and miniproteins can effectively target c-MYC by either disrupting critical protein–protein interactions or directly blocking DNA binding (Table 1), highlighting their potential in overcoming the challenges associated with this historically undruggable transcription factor.

## 3. Deep Learning Architectures in Peptide Drug Discovery

### 3.1. Data Representation & Geometric Deep Learning

**Figure 4:**
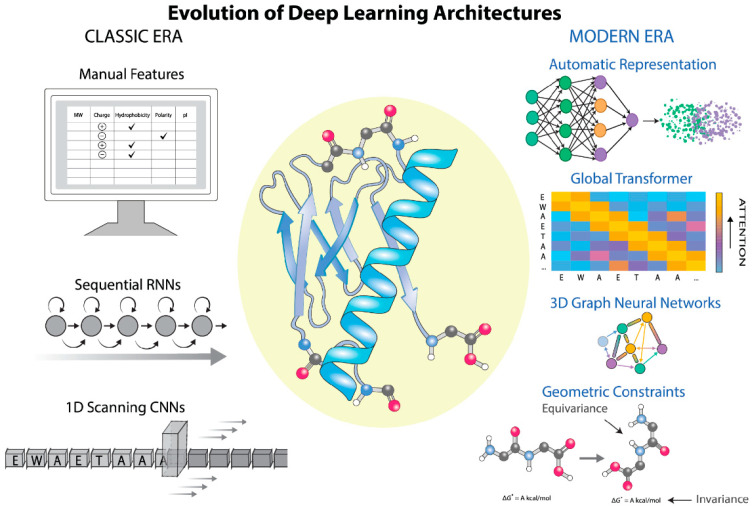
Evolution of deep learning architectures in protein science: From manual feature engineering to geometric and transformer-based representations. RNNs: Recurrent Neural Networks. CNNs: Convolutional Neural Networks.

To effectively target undruggable proteins with shallow surfaces, AI must transcend simple sequence modeling. This section outlines the shift toward Geometric Deep Learning, which integrates 3D spatial constraints and physical symmetries. The following discussion clarifies the fundamental purposes of various architectures, establishing a conceptual framework for the specific models presented in subsequent sections (Figure 4). By understanding these architectural foundations, we can better appreciate how they enable AI to design therapeutic peptides with atomic-level precision while strictly adhering to fundamental biological and physical laws.

The evolution of deep learning architectures has shifted the research focus from the utilization of manual features to the capacity for automatically learning complex representations from raw data [63,64,65]. In peptide design, initial sequence-based models leverage Recurrent Neural Networks to process amino acids sequentially, which helps capture contextual dependencies through hidden states [66]. However, thoroughly addressing the problem of vanishing gradients across long sequences remains a major challenge. This phenomenon hinders the ability of model to capture distant relationships between amino acids, thereby disrupting the preservation of spatial interactions necessary for protein inhibition. To overcome this, variants such as Long Short-Term Memory (LSTM) and Gated Recurrent Units (GRU) have been implemented, utilizing sophisticated gating mechanisms to maintain signal stability [64]. A significant turning point occurred with the emergence of the Transformer architecture, a system that eliminates sequential processing to establish global connections through the Self-attention mechanism. The capacity for multidimensional parallel computation and the capture of long-range dependencies allow the Transformer to provide a solid foundation for modern variants within protein structure research [67].

In parallel with sequence models, Convolutional Neural Networks approach peptides as 1D image processing objects, where kernels function as scanning mechanisms to automatically extract structural motifs and recognize complex synergistic relationships between amino acids that cannot be described by ordinary linear additive calculations [68]. This method enables parameter optimization through backpropagation to detect intricate physicochemical features without the need for manual definition [69]. Regarding molecular graph representations, Message-Passing Neural Networks have established a systemic shift by allowing atoms to continuously update states based on information from neighboring nodes [70,71]. The combination of message-passing neural networks and newer Graph Transformer models has expanded the focus from local to global contexts, allowing all atoms to interact directly regardless of bonding distance while still preserving core molecular topological properties through shortest path distance encodings [70,72,73].

To ensure that Artificial Intelligence models strictly adhere to physical laws, geometric constraints are integrated as essential inductive biases. These constraints are primarily characterized by the principles of invariance and equivariance, which dictate how a model responds to spatial transformations. Invariance ensures that scalar properties such as free energy or binding affinity maintain consistency regardless of rotations or translations in space. Meanwhile, Equivariance preserves the directional information of vectors, ensuring that outputs such as atomic coordinates or interaction forces transform correspondingly with the input [74]. The application of these symmetry principles not only optimizes computational resources by removing the requirement for data augmentation but also enhances generalization capabilities, allowing the model to focus on core geometric features instead of being influenced by arbitrary coordinate systems [72,74,75,76].

### 3.2. Generative Scaffold Design

**Figure 5:**
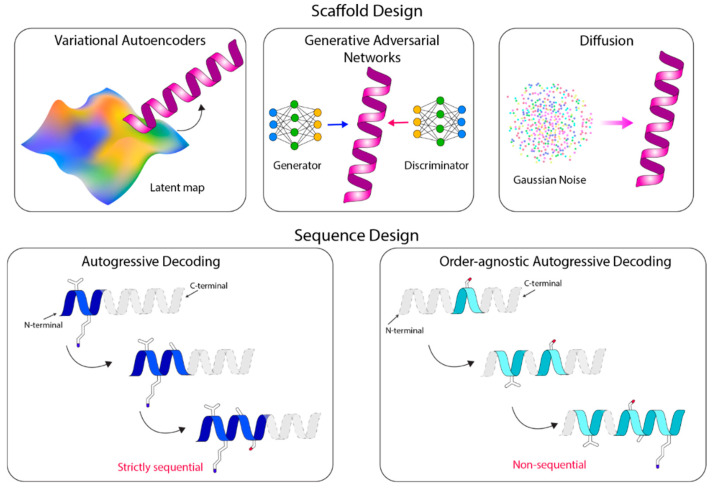
Overview of Generative and Autoregressive models for de novo peptide design.

Beyond static data representation, the emergence of generative architectures has revolutionized peptide design by projecting discrete molecular information into continuous mathematical spaces (Figure 5). This transition enables the systematic optimization and de novo synthesis of novel sequences, moving the field from mere structural prediction toward the intentional engineering of functional biomolecules.

Deep learning architectures have initiated a new era in peptide design by transforming discrete molecular data into mathematical spaces that allow for optimization. Within this context, Variational Autoencoders (VAEs) play a crucial role in compressing SMILES character sequences into a continuous and multidimensional latent space through the use of an encoder and a decoder. The advantage of VAEs lies in their probabilistic nature, which helps organize the latent space into a structural map where physicochemical properties are distributed according to defined gradients. The existence of these property gradients enables the system to precisely calculate the direction for coordinate adjustment to guide an existing structure toward regions of space with higher performance instead of relying on random trials. Despite these advantages, VAEs often suffer from a phenomenon known as posterior collapse, where the model ignores certain latent dimensions, leading to a loss of structural diversity. Furthermore, the objective function of VAEs tends to favor average probability distributions, which frequently results in blurry molecular outputs. In the context of peptide design, this lack of precision can produce structures with unrealistic bond lengths or suboptimal side chain orientations, requiring extensive refinement to achieve biological viability [77,78].

In parallel, Generative Adversarial Networks (GANs) establish a mathematical framework based on the opposition between a generator network and a discriminator network. Rather than performing individual optimization, GANs reach a state of dynamic equilibrium that facilitates the efficient and rapid generation of data samples simulating the actual distribution. The primary challenge with GANs involves the inherent difficulty of maintaining a balance between the generator and the discriminator during training. This often leads to mode collapse, a state where the model produces a limited variety of peptide sequences that happen to fool the discriminator rather than exploring the full chemical space. Additionally, because GANs do not explicitly model the probability density of the data, they provide less control over the specific physical properties of the generated molecules compared to more recent approaches [79,80,81,82].

The emergence of Diffusion models has established a new standard, surpassing the limitations of VAEs and GANs in processing complex biological systems. This process operates based on two stages, consisting of forward diffusion to introduce noise into the data and reverse diffusion to recover information from a chaotic state [65,82]. Through theoretical frameworks such as denoising diffusion probabilistic models (DDPMs), noise conditional score networks (NCSNs), and stochastic differential equations (SDEs), the model can control the convergence process from Gaussian noise into protein structures with atomic-level precision [65,82,83,84]. The application of stochastic differential equations also provides a technical advantage by allowing the use of advanced numerical solvers to flexibly adjust between the speed of peptide library screening and the accuracy of configurational refinement [82,84].

RFdiffusion marks a significant advancement by transforming the problem of static structure prediction into a process of dynamic generation based on diffusion models. Instead of being limited by sequences existing in nature, RFdiffusion initiates from an entirely amorphous cloud of Gaussian noise and gradually shapes new protein backbones capable of stable folding through the use of an SE(3)-equivariant denoising algorithm, which handles rotation and translation simultaneously. This characteristic ensures independence from a reference frame, allowing the model to describe precise three-dimensional structures without dependence on a global coordinate system. Furthermore, the integration of Self-attention layers from RoseTTAFold alongside Rigid frame Representation enables RFdiffusion to define each residue through alpha carbon coordinates and the orientation of the peptide backbone atoms (N, C_α_, C) instead of processing discrete atoms. This approach allows for the accurate calculation of interactions between amino acids distant in space as they converge toward an optimal configuration. The self-conditioning mechanism functions as a feedback loop, helping the model refine internal predictions through each reverse diffusion step, thereby producing superior geometric consistency. Due to diffusion dynamics, RFdiffusion can reverse engineer complex structures such as binders adhering to functional hotspots or construct scaffolds surrounding functional motifs with atomic-level accuracy [85].

### 3.3. Sequence Design

Once the generative models have established the optimal backbone architecture for the peptide, the subsequent crucial step involves determining the specific amino acid sequence that ensures structural stability. This sequence design process serves to translate the generated 3D blueprints into functional biomolecules through sophisticated decoding mechanisms.

Autoregressive decoding mechanisms serve as a fundamental basis in modern protein language models, transforming the challenge of sequence design into a step-by-step probability estimation process. The core principle of this framework is the prediction of the next amino acid based on the entire context of previously generated elements [86,87,88]. However, this mechanism faces a significant challenge regarding the accumulation of errors, as a suboptimal prediction at an early stage can introduce noise and distort the entire subsequent decoding process [89]. The fixed unidirectional nature of decoding also results in a lack of global context and creates difficulties in capturing complex spatial interactions that determine the stability of the 3D structure [72,86,89].

To address the limitations discussed in the preceding section, ProteinMPNN introduced a mechanism known as Order-agnostic Autoregressive Decoding. By training on random permutations of all residue positions, the model enables the selection of any decoding sequence during the inference process. This advancement is particularly significant because it permits design around existing functional regions, such as enzyme active sites, by incorporating them into the context before decoding the remaining segments [72,90,91].

The core strength of ProteinMPNN lies in the representation of protein structures as a sparse residue graph. Within this network, each amino acid functions as a node, connecting only to 32 to 48 nearest neighbor nodes based on the distance between C_α_ atoms. Instead of utilizing raw coordinates, which are susceptible to error, the model employs a message-passing neural network algorithm to extract invariant geometric features. A primary breakthrough involves the simultaneous update of both node and edge features, integrated with the encoding of distances between backbone atoms including N, C_α_, C, and O, alongside a virtual C_β_ atom. This approach establishes a superior inductive bias, enabling the model to learn the optimal side chain packing patterns observed in natural proteins [72].

Furthermore, ProteinMPNN redefines the design procedure through a mechanism known as Order-agnostic Autoregressive Decoding. Rather than being restricted by fixed decoding from the amino terminus to the carboxyl terminus as observed in classical models, ProteinMPNN undergoes training on random permutations of every residue position. This characteristic enables users to actively lock essential functional regions, allowing the model to freely design the surrounding sequence while maintaining global consistency. Finally, to adapt to empirical data, the model enhances its robustness to noise by incorporating Gaussian noise into the backbone coordinates during the training phase. Consequently, ProteinMPNN performs effectively not only on perfect crystal structures but also remains highly efficient when processing backbones predicted by computational tools such as AlphaFold2 [72,91].

### 3.4. Structure Validation

Following the design of sequences via ProteinMPNN, the utilization of advanced structure prediction models, such as AlphaFold models, constitutes an essential validation step to verify the folding capacity and stability of de novo designs.

Within the AlphaFold2 architecture, the Evoformer serves as the primary component and functions as a sophisticated encoder that transforms raw data into complex evolutionary and geometric features. This mechanism facilitates a continuous exchange of information between the Multiple Sequence Alignment representation and the pair representation [92,93]. By employing pathways such as gated self-attention and triangular multiplicative updates, the Evoformer enables the model to capture long-range dependencies between residues that are distant in sequence while enforcing triangular inequality within the protein geometry [92]. Due to its capacity for structure prediction with atomic-level accuracy, the Evoformer has revolutionized structural biology, drug discovery, and de novo protein design [92,93].

However, the Evoformer architecture possesses significant technical barriers. In principle, the model primarily predicts a single static state, which results in difficulties when accounting for conformational diversity [92,93]. Furthermore, the Evoformer relies excessively on coevolutionary signals derived from Multiple Sequence Alignment, leading to suboptimal performance for orphan proteins or artificial protein sequences [92]. Most notably, the model lacks sensitivity to point mutations [94] and overlooks critical chemical contexts such as the presence of ligands, metal ions, or post-translational modifications [92,93]. These limitations reduce the applicability of AlphaFold2 within more realistic and complex biological systems.

The introduction of AlphaFold 3 addresses several limitations of the previous version by expanding the modeling scope into a unified framework. Rather than focusing exclusively on proteins, AlphaFold 3 possesses the capacity to simultaneously predict the structures of complexes, including nucleic acids such as DNA and RNA, ligands, and ions with high precision. Notably, AlphaFold 3 significantly improves the prediction of interactions between antigens and antibodies while reducing the dependence on Multiple Sequence Alignment (MSA), which enhances performance in single sequence mode. This development facilitates the study of rare protein families and the design of novel biological molecules [95,96].

The revolutionary advancement of AlphaFold 3 lies in the replacement of the Evoformer with the Pairformer and the integration of a Diffusion Module. The Pairformer serves as the core component for data processing, which significantly simplifies the handling of MSA and focuses on pair representation to maintain geometric consistency. Subsequently, instead of utilizing a decoder based on dihedral angles as seen in AlphaFold2, AlphaFold 3 employs a Diffusion Module to directly predict raw atomic coordinates from noisy data. Through a multiple-scale denoising mechanism, the model can sharply determine both local stereochemical details and global structures without the requirement for complex reference frames. The synergy between the interaction blueprint provided by the Pairformer and the generative capacity of the diffusion model enables AlphaFold 3 to eliminate rigid loss functions, resulting in a flexible and robust system for resolving complex biomolecular structures [95,96].

### 3.5. Predictive Models for ADMET and Property Optimization

The optimization of absorption, distribution, metabolism, excretion, and toxicity (ADMET) profiles constitutes a decisive screening step for transitioning a peptide candidate from theoretical design to clinical application. Unlike conventional small molecules, peptide therapeutics inherently suffer from poor proteolytic stability and limited passive membrane permeability due to their large molecular weight and hydrophilic backbones.

To efficiently navigate the vast chemical space of macrocycles violating Lipinski’s Rule of 5, modern computational drug discovery has moved beyond traditional, static quantitative structure–activity relationship (QSAR) methods toward a stratified hybrid screening paradigm [97]. This overarching approach leverages the high-throughput capabilities of deep learning to rapidly filter vast candidate libraries, while strategically integrating physics-based simulations to ensure thermodynamic accuracy [77]. Rather than relying on isolated regression or classification boundaries, contemporary frameworks utilize Multi-task learning networks to evaluate critical biological indices, such as solubility, systemic toxicity, and proteolytic susceptibility [98].

Within this predictive pipeline, a two-tier screening strategy can be operationalized to systematically optimize both prediction accuracy and computational expenditures. The front-end tier deploys explainable Geometric Deep Learning and Message-Passing Neural Networks (MPNNs) to scan broad structural landscapes, predicting chameleonic behavior and mapping enzymatic cleavage sites to guide sequence stabilization [99]. Subsequently, the back-end tier reserves computationally intensive, enhanced-sampling Molecular Dynamics (MD) simulations strictly for high-potential shortlists. This targeted biophysical validation precisely computes the thermodynamic boundaries and free-energy profiles of conformational transitions, ensuring that final candidates possess optimal protease stability and membrane permeability before advancing to expensive in vitro testing.

As AI and deep learning technologies continue to advance and become increasingly integrated, their applications in de novo peptide design are expanding. An overview of modern AI-driven tools and architectures specialized for de novo peptide design is summarized in Table 2.

## 4. Integrated De Novo Peptide Design Workflows: A Promising Paradigm for Targeting Undruggable Proteins

### 4.1. Peptide Scaffold Design via RF Diffusion

The application of RFdiffusion in de novo peptide design could potentially enable the generation of entirely novel backbones that do not rely on templates existing in nature. We envision that this proposed process could be structured based on three core coordination strategies to optimize interaction capabilities with complex protein surfaces.

#### 4.1.1. Target Context Scaffolding Strategies

To address the challenges presented by the flat interaction surfaces and the absence of deep binding pockets on KRAS, the proposed workflow suggests applying a strategy of constructing scaffolds directly within the 3D space surrounding the target protein. Rather than designing peptides in isolation, our envisioned framework seeks to enable the RFdiffusion model to automatically adjust scaffold morphology based on the analysis of the chemical surface of the target to establish new contact points. A typical demonstration of this capability was recorded for the MDM2 p53 complex, where the target context scaffolding method helped increase the average buried surface area by 31%, thereby elevating the binding affinity to picomolar levels and making it over 1000 times stronger than the natural peptide [85].

For KRAS, the optimization of scaffold shape to cover large surface areas at the Switch I and Switch II regions is anticipated to be the key suggestion to achieving interaction forces strong enough to inhibit intracellular signaling pathways [105,106]. For intrinsically disordered proteins such as c-MYC, we propose that RFdiffusion can facilitate the simulation of transient intermediate structural states, potentially allowing the peptide framework to stabilize the transition from an unstructured form to a functional folded conformation upon binding [107].

#### 4.1.2. Navigating Design via Interface Hotspots

A core component proposed within this workflow is the utilization of interface hotspots as a navigation system for the reverse diffusion process. In this conceptual framework, the RFdiffusion algorithm would use critical amino acid residues on the target surface as guideposts to configure the binding position of the peptide into specific binding pockets that are not the natural priorities of the model. The efficacy of this mechanism was confirmed through the design of binders for the IL-7Rα receptor, where providing information regarding hotspots at site 2 navigated the peptide to bind precisely at the desired area instead of focusing on site 1 as a default [85].

This navigation capability is extremely important for KRAS, as it can be leveraged for peptide design with high specificity for individual mutation types such as G12C or G12D by directly targeting cryptic pockets recently discovered near the Switch II region [105]. Regarding MDM2, the residues Ile61 and Gly58 are identified as strategic hotspots to guide the design model focusing on the hydrophobic pocket of p53 [52]. For c-MYC, the orientation process focuses specifically on the segment labeled as LC46 encompassing residues 370 to 414 within the bHLHZip domain, which is the region mainly responsible for interacting with the partner Max [107].

#### 4.1.3. Structural Configuration via Fold Conditioning

To ensure structural stability and practical applicability, the proposed workflow envisions applying a fold conditioning mechanism to shape the peptide into defined geometries, such as alpha-helical bundles, from the initial stage. Studies on insulin and TrkA receptors demonstrate that the application of fold conditioning helped generate structurally stable binders with binding affinities reaching nanomolar levels from the very first trials [85].

Regarding the KRAS and MDM2 target, the selection of an alpha helical structure is expected to not only facilitate the mimicry of the interaction modes of natural effector proteins, but also achieve stable occupancy of deep hydrophobic pockets and provide an advantage for experimental synthesis [106,108]. By pre-defining these structural constraints, the model could potentially narrow the vast conformational search space, ensuring that the generated backbones possess the necessary rigidity to maintain their bioactive shape upon binding. Furthermore, this structural “scaffolding” is anticipated to minimize the entropic penalty typically associated with the folding of flexible peptides, thereby significantly enhancing the overall therapeutic potential of the designed inhibitors.

The combination of this stable structural scaffold and the sequence design algorithm of ProteinMPNN is projected to optimize side chain packing, which would allow researchers to actively apply stabilization techniques such as peptide stapling to maintain the functional configuration of the peptide.

### 4.2. Sequence and Physicochemical Property Optimization via Proteinmpnn

Within the proposed workflow targeting undruggable proteins, we project that the sequence design stage would play a decisive role in transforming the structural backbones from RFdiffusion into functional biological entities. Rather than relying on traditional physical energy functions, which frequently encounter errors when processing surfaces with high flexibility, this process prioritizes the use of ProteinMPNN.

#### 4.2.1. Shifting Toward Graph Neural Networks

As we envision its application here, this tool would operate based on the power of Graph Neural Networks to decode protein architectural rules directly from experimental data within the Protein Data Bank. The replacement of complex thermodynamic calculations with the learning of amino acid probability distributions allows ProteinMPNN to outperform older methods such as Rosetta in both computational speed and experimental accuracy, particularly in ensuring the solubility and precise folding capacity of the peptide [72].

#### 4.2.2. Order-Agnostic Autoregressive Decoding Mechanisms

The pivotal point conceived in the designed framework is the application of an Order-agnostic Autoregressive Decoding mechanism. Unlike traditional protein language models restricted by linear sequence design from the amino terminus to the carboxyl terminus, ProteinMPNN could allow researchers to actively lock critical structural motifs at fixed positions.

This capability was experimentally demonstrated in the design of binders for the SH3 domain of the Grb2 protein. By fixing the target binding motif consisting of the proline-rich sequence and redesigning the entire surrounding sequence, ProteinMPNN generated binders with strong binding signals and high specificity on biolayer interferometry instruments, far surpassing previous designs from Rosetta, which had failed in folding or binding [72].

In the context of peptide design for KRAS, we suggest this strategy can be utilized to facilitate the complete preservation of key amino acids that interact directly with the Switch I and Switch II pockets [106], while the model freely optimizes the remaining parts of the scaffold to achieve maximum geometric and physicochemical complementarity.

For systems requiring the preservation of native interactions such as MDM2/p53, ProteinMPNN can be configured to fix the side chain positions corresponding to Phe19, Trp23, and Leu26 [52]. Restricting these specific residues is expected to allow the model to concentrate on optimizing the remaining portions of the surrounding sequence, aiming to generate Van der Waals interaction networks and hydrophobic contacts that are more stable than those found in the natural peptide.

Particularly for intrinsically disordered proteins such as c-MYC, the role of ProteinMPNN would hypothetically shift toward optimizing amino acid probabilities to enhance structural stabilization. By populating the LC46 segment with residues that have a high propensity for alpha-helix formation [107], the model is anticipated to help minimize the entropic cost incurred when the peptide transitions from a free state to a functional folded conformation. This process is designed to yield a sequence characterized by structural preorganization, which could significantly increase the efficiency of inhibiting heterodimer formation with Max.

#### 4.2.3. Surface Property Optimization

Regarding difficultly to target protein with shallow surfaces and high levels of hydrophobicity, such as KRAS and MDM2, ProteinMPNN is proposed to provide an automated solution to address obstacles involving stability and solubility through the optimization of amino acid residues exposed to the solvent. By applying backbone noise during the training process, the model is expected to focus on global structural features instead of local errors, ensuring the designed peptide possesses high thermal stability and accurate folding configurations [72]. This workflow would allow for the strategic redistribution of polar amino acids on the peptide surface, aiming to transform binding pockets previously considered inaccessible into target sites that can be occupied by specialized designed proteins. The combination of preserving core interactions and the flexible restructuring of the support framework is envisioned as the key to generating new generations of binders with superior affinity for mutant variants of KRAS and other undruggable targets.

### 4.3. Structural Validation and Interaction Prediction via AlphaFold 3

Within the workflow for peptide design, the generation of structural backbones via RFdiffusion and the optimization of sequences through ProteinMPNN represent only the initial stage; the true challenge lies in validating whether these designs can bind precisely to shallow binding pockets or allosteric sites.

#### 4.3.1. Transitioning Toward the Atomic Diffusion Module

To address this issue, AlphaFold 3 is proposed as a core validation tool due to the revolutionary shift from the Evoformer architecture to a diffusion module. Unlike its predecessor, which relied on torsion angles and evolutionary signals, AlphaFold 3 operates directly on raw atomic coordinates using a multiple-scale denoising mechanism which is designed to move atoms from a random state to precise spatial positions [95]. This shift is expected to enhance the model capability to predict complex systems, including proteins, ligands, and ions, with atomic-level accuracy. Consequently, it is expected to offer an enhanced framework for simulating the hydrogen bonding networks and hydrophobic interactions between the designed peptide and mutant amino acids, such as G12D or G12V on KRAS.

#### 4.3.2. Blind Docking Strategy and Dynamic Binding Pocket Discovery

The breakthrough of AlphaFold 3 within the proposed framework is envisioned to leverage a blind docking strategy, allowing for the scanning of the entire protein surface to identify interactions without prior structural information regarding the binding pocket.

This capability was experimentally demonstrated as AlphaFold 3 precisely identified the allosteric binding position on the PI5P4Kγ enzyme with an impressive root mean square deviation of only 0.37 Å, a location that traditional methods frequently overlook. Furthermore, the reliability of the model was confirmed through the structural prediction of the ternary complex of the PORCN receptor bound simultaneously with an inhibitor and the WNT3A peptide, achieving a root mean square deviation value of 1.00 Å [95].

For a target previously considered undruggable due to smooth surfaces, such as KRAS, the joint prediction mechanism of AlphaFold 3 is anticipated to facilitate the simulation of how protein structures change shape through induced fit upon contact with a peptide, thereby potentially uncovering temporary binding pockets or new allosteric sites [106].

In the validation of designs for the complex involving MDM2/p53, AlphaFold 3 is proposed to enable the verification of geometric accuracy as key amino acid residues such as Phe19, Trp23, and Leu26 penetrate deeply into the hydrophobic pockets at the N-terminal domain of MDM2 [52]. This simulation process is anticipated to be critical in confirming whether the designed peptide successfully reproduces the characteristic alpha helical structure of p53 to occupy the active cavity stably.

Furthermore, regarding an intrinsically disordered protein such as c-MYC, AlphaFold 3 could offer a significant advantage in simulating the structural transition from a flexible state to a tightly folded conformation when the peptide interacts with the LC46 segment within the bHLHZip domain [107].

#### 4.3.3. Effective Virtual Screening Based on the ipTM Confidence Metric

Rather than performing costly physical screening experiments, the proposed workflow suggests leveraging the interface predicted template modeling score of AlphaFold 3 as a potential filter to rank peptide candidates. Studies indicate that when focusing on a specific binding pocket, the success rate of the model on the PoseBusters V1 dataset significantly increase from 76.4% to 90.2%, demonstrating the capacity for structural refinement and extreme reliability in decoding complex atomic interactions [95]. Candidates possessing high interface confidence scores and tight geometric complementarity will receive priority for experimental validation to ensure the optimization of binding affinity and the feasibility of AI-driven design strategies. The combination of the ability to decode dynamic binding pockets and the refinement of affinity by AlphaFold 3 is expected to establish a solid foundation to transform theoretical designs into specific peptide therapies.

### 4.4. Evaluation of Physicochemical Properties and Bioavailability

To ensure that the designed peptides targeting undruggable proteins function effectively within the intracellular environment, the workflow suggests implementing the stratified hybrid ADMET paradigm detailed in Section 3.5. Rather than executing resource-intensive biophysical computations across an entire library, a two-tier screening workflow is envisioned to systematically balance prediction throughput with thermodynamic accuracy.

In the initial high-throughput phase, sequence-based attention models (e.g., DeepCleave) could be deployed to rapidly map proteolytic susceptibility and identify enzymatic cleavage sites across thousands of generated sequences. This rapid filtering allows for the strategic identification of vulnerable structural hotspots, guiding the precise introduction of backbone cyclization or non-natural amino acid substitutions to maximize metabolic half-life [109].

Concurrently, geometric neural filters could evaluate global surface features to prioritize macrocyclic candidates based on their predicted capacity for dynamic chameleonic masking. In the subsequent low-throughput phase, these top-ranked AI-flagged candidates would be subjected to targeted, enhanced-sampling Molecular Dynamics (MD) simulations. This focused biophysical validation is envisioned to precisely calculate structural transition barriers and membrane partitioning free energy with the aim of mitigating the accumulation of predictive errors inherent in black-box neural architectures [99]. This proposed screening framework could be tailored specifically to the unique structural and spatial demands of the target proteins highlighted in this study.

For mutant variants of KRAS (such as G12D or G12V), geometric models would first be deployed to screen for macrocyclic backbones capable of shielding polar groups around the shallow Switch I and Switch II regions. Focused REST-MD simulations would then follow to verify the thermodynamic feasibility of this chameleonic transition, ensuring that the selected candidate peptides possess the physical capacity to cross the lipid bilayer and occupy these intracellular cryptic pockets [99].

Finally, for the intrinsically disordered c-MYC protein, computational deep mutational scanning could prioritize sequences with a high propensity for alpha-helix preorganization within the LC46 segment [107]. This targeted design is anticipated to minimize the entropic penalty typically incurred during the flexible-to-folded structural transition, while localized neural predictors simultaneously ensure the motif remains highly resistant to rapid degradation by circulating proteases. Consequently, this integrated, stratified paradigm is anticipated to efficiently deliver optimized peptide candidates that possess both robust metabolic stability and cellular permeability prior to experimental validation.

**Figure 6:**
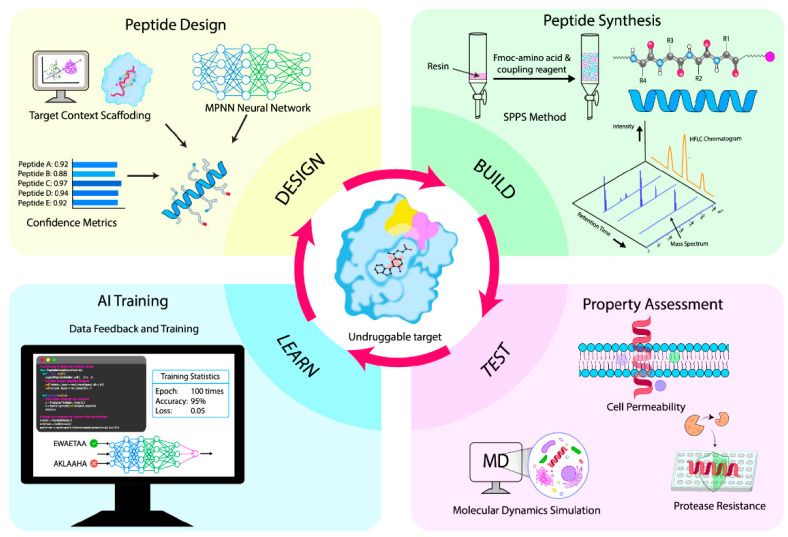
An iterative AI-experimental framework for the discovery of bioactive peptides against undruggable targets. MPNN: Message-Passing Neural Network; SPPS: Solid Phase Peptide Synthesis; HPLC: High-Performance Liquid Chromatography.

In conclusion, while this section outlines the conceptual steps for integrating these computational tools into a theoretical peptide design workflow, it is important to note that the practical execution of such a pipeline faces clear technical and biological limitations (Figure 6, Table 3). A detailed analysis of these methodological bottlenecks, structural constraints, and subsequent pharmacological challenges is presented in Section 5.

## 5. Challenges and Limitations

While AI-based peptide engineering represents a promising avenue for targeting historically undruggable systems, the successful translation of AI-designed peptides into clinically viable therapeutics remains associated with numerous challenges. These challenges arise from both the limitations of current AI frameworks and the inherent drawbacks of peptide therapeutics.

Peptide-based therapeutics targeting undruggable proteins still face significant challenges related to cellular delivery, stability, and pharmacokinetics. Because many of these targets are intracellular proteins, therapeutic agents must efficiently cross the lipid bilayer to reach the cytosol. Unlike small molecules, peptides generally exhibit poor membrane permeability and are unable to readily diffuse across cellular membranes. Even when internalized, typically via macropinocytosis, peptides are frequently sequestered within endosomal compartments and fail to access their cytosolic targets [110]. To improve cellular uptake, poly-arginine tags, also known as cell-penetrating peptides, are often appended. However, this approach introduces safety concerns, as highly cationic peptides are associated with mast cell degranulation and histamine release, which may lead to severe adverse effects, including anaphylactic shock [111].

In addition, peptides are inherently susceptible to rapid degradation by proteases and peptidases present in the gastrointestinal tract, blood circulation, and intracellular environment. Linear peptides are particularly unstable; for instance, the prototype Raf-1 peptide targeting KRAS undergoes near-complete degradation in human serum within 24 h. Consequently, chemical modifications such as peptide stapling and cyclization are commonly employed to enhance structural stability [26]. Peptides also typically display suboptimal pharmacokinetic properties, including rapid clearance via hepatic and renal pathways, resulting in short circulation half-lives [29].

On the other hand, the application of artificial intelligence in peptide-based inhibitor development introduces additional structural, technical, and pharmacological challenges. From a structural perspective, peptides are intrinsically flexible, making it difficult to accurately predict their bound conformations and associated conformational transitions. This flexibility imposes a significant entropic penalty upon binding, which remains challenging for current computational models to quantify [8]. Moreover, most AI-based structural prediction tools generate static representations and fail to capture the dynamic conformational ensembles that are essential for realistic modeling of protein–peptide interactions. This limitation is particularly pronounced for intrinsically disordered or highly flexible targets, where static models may not reflect biologically relevant states [95,103].

From a technical standpoint, AI hallucination remains an important challenge in peptide and protein design. The “hallucination” approach, also known as activation maximization, uses structure-prediction networks such as AlphaFold2 and RoseTTAFold in reverse to generate sequences predicted to fold into highly confident structures, based on metrics such as pLDDT and pAE. Although this strategy enables the design of high-affinity binders without predefined geometric constraints, it may also generate “adversarial sequences” that achieve high confidence scores but fail experimentally because of instability, insolubility, or incorrect folding and binding behavior. In addition, AI systems may produce spurious structural order by generating compact structures in regions that should remain intrinsically disordered. To address these limitations, diffusion-based approaches such as RFdiffusion have emerged as more robust alternatives, while redesigning hallucinated backbones using ProteinMPNN may further improve sequence quality and experimental feasibility. Furthermore, experimental validation techniques such as circular dichroism spectroscopy and structure-resolution pull-down assays remain essential for verifying that hallucinated binders possess the predicted structural and biophysical properties [8,72,85]. Compounding these challenges, several limitations associated with current AI-driven prediction and scoring frameworks may further compromise the reliability of the generated outputs. Even advanced models such as AlphaFold 3 are not free from structural inaccuracies, including violations of stereochemistry and atomic clashes, particularly in complex systems [95]. Commonly used scoring functions, such as docking energies, often fail to fully capture solvent effects and molecular dynamics, limiting their reliability as predictors of true binding affinity [102].

Data availability and quality represent another major limitation. Although structural databases are extensive, high-quality experimental data on peptide–protein interactions remain scarce. As of recent estimates, only a small fraction of peptide-containing structures provide detailed interaction information. In addition, available datasets are often biased toward positive results and natural amino acids, with limited representation of failed designs or non-canonical modifications. This bias is particularly problematic for synthetic peptides, which frequently involve macrocyclic structures and non-natural amino acids that are not well supported by existing computational tools. As a result, modeling such systems often requires manual intervention or specialized workflows, reducing efficiency and scalability. Moreover, the lack of iterative feedback between computational predictions and experimental validation (dry–wet loops) allows prediction errors to accumulate over successive design cycles. Experimental data are also typically generated under narrow conditions, limiting their generalizability to diverse physiological environments [77].

In addition to these scientific challenges, practical limitations must also be considered. The computational cost associated with advanced AI-driven design such as generative modeling or multi-generation optimization algorithms can be substantial, often requiring extensive GPU (Graphics Processing Unit) resources and long processing times to generate a limited number of viable candidates [100].

A key issue is the disconnect between predicted binding affinity and actual therapeutic efficacy, as high affinity alone does not ensure selectivity or minimize off-target effects. Predicting critical drug-like properties, including solubility, immunogenicity, toxicity, metabolic stability, and half-life, remains highly challenging. Membrane permeability, in particular, is difficult to model because of the “chameleon-like” behavior of peptides, which adopt different conformations in aqueous and lipid environments, an effect that static computational models fail to capture [101]. Immunogenicity in peptide therapeutics refers to unintended or adverse immune responses that may be triggered either by the active peptide sequence itself or by impurities introduced during manufacturing and formulation. These immune responses generally involve T-cell and B-cell activation, leading to the production of anti-drug antibodies (ADAs), which can neutralize therapeutic efficacy, alter drug clearance, and potentially cause serious adverse effects, including hypersensitivity, anaphylaxis, and autoimmunity. Therefore, the integration of appropriate filtering and evaluation strategies into AI-based design frameworks is essential for improving the safety and therapeutic efficacy of designed peptides [112,113].

Finally, when designing peptide therapeutics using AI, considerations related to manufacturing cost and scalability under GMP conditions are also critically important. Scaling up peptide production to GMP standards involves substantial material costs due to the large excesses of reagents required in solid-phase peptide synthesis, as well as the energy-intensive nature of traditional isolation methods such as lyophilization. Scalability is further hindered by manufacturing risks and low yields, particularly for peptides longer than 30 amino acids, for which failure rates are estimated to reach approximately 20% [114,115,116]. Although several approaches, including hybrid SPPS/LPPS strategies, microwave-assisted technologies, and the use of nanofiltration or spray-drying methods, have been introduced to reduce production costs [114,116], peptide design must still carefully balance therapeutic performance with manufacturing feasibility and economic efficiency.

## 6. Future Perspectives

Despite persistent challenges, peptide-based strategies represent a promising avenue for targeting historically undruggable proteins, as evidenced by the emergence of cyclic and stapled peptides in clinical trials. In particular, recent advances in deep learning and related computational approaches have the potential to substantially accelerate peptide design and optimization.

Nevertheless, the effectiveness of AI-driven peptide design remains critically dependent on the quality of training data. Models trained on biased or limited datasets may generate predictions that fail to translate into experimental systems. Therefore, careful dataset preparation and the expansion of high-quality training data are essential for robust learning and improved predictive performance. The integration of experimentally derived datasets, including binding affinity, toxicity, and pharmacokinetic profiles, can further enhance the performance of generative models. In this context, active learning frameworks, in which experimental results are iteratively incorporated back into the training process, represent a powerful strategy for continuously refining predictions and reducing downstream failure rates [103]. Moreover, emerging reasoning-based AI systems offer an additional promising direction, as they are capable not only of generating peptide sequences but also of providing mechanistic explanations for their design decisions [117].

Furthermore, most AI-based structural prediction tools predominantly generate static representations. Recently, MD simulations have been widely applied to evaluate binding stability, estimate free energies, validate mutant selectivity, and predict membrane accessibility and permeability [100,103]. The integration of artificial intelligence with molecular dynamics (MD) simulations therefore represents a promising strategy for gaining insights into structural stability and conformational dynamics. Together, establishing an iterative workflow that links training data, computational design, and experimental validation is essential not only for generating promising peptide candidates, but also for expanding the design space while maintaining the reliability of AI-based tools.

Beyond improvements in training data, the continued development of computational tools will further broaden the scope of peptide discovery. Although many current computational approaches remain optimized for natural amino acid sequences, increasing efforts are being directed toward the incorporation of non-natural amino acids and the design of stapled and cyclic peptides. The development of dedicated tools in this area could substantially expand the accessible design space and enable the generation of peptides with improved stability and in vivo performance [118,119]. Such developments are particularly important because structural modification strategies are increasingly employed to address major limitations of peptide therapeutics, including conformational flexibility, susceptibility to proteolytic degradation, and poor membrane permeability.

In parallel, quantum computing is expected to further accelerate the development of computer-aided drug discovery. This technology may help overcome several limitations of classical computing systems when processing extremely large and complex datasets. For example, quantum computing integrated with generative AI frameworks may facilitate the exploration of vast chemical spaces that remain computationally challenging for conventional systems, thereby enabling the design and ranking of millions of candidate KRAS inhibitors [120]. Meanwhile, emerging hybrid frameworks that integrate quantum chemistry-derived potentials into AI pipelines may help eliminate hallucinated or biophysically unrealistic conformational states generated by AI systems [77]. In addition, specialized methods based on quantum computing can identify protein interface “hot spots” with a level of chemical detail that is difficult to achieve using conventional molecular mechanics [3].

Successful peptide design is not defined solely by computational prediction or laboratory validation, but ultimately by clinical translation. Bridging this gap requires the integration of advanced technologies across multiple stages of development, ranging from computational design and compound screening to formulation and drug delivery. The combination of computational design platforms with advanced experimental screening approaches, such as phage display and affinity selection-mass spectrometry, can substantially accelerate the discovery pipeline while reducing both cost and resource consumption. In parallel, advances in formulation technologies play a critical role in improving the pharmacokinetic properties of peptide therapeutics. Strategies including nanoparticle-based delivery systems and microneedle technologies have demonstrated potential for overcoming limitations in biological stability and enhancing targeted delivery of peptide drugs [121,122].

Furthermore, the therapeutic potential of peptides continues to expand. Therapeutic peptides are increasingly being explored in combination with small molecules and immunotherapies. Combination strategies seek to leverage the high specificity and favorable safety profiles of peptides to enhance the efficacy of both conventional treatments, such as chemotherapy, and emerging therapeutic modalities, including immunotherapy. One important example is peptide–drug conjugates (PDCs), in which peptides function as targeting moieties that selectively deliver cytotoxic small-molecule payloads to diseased cells [123]. Another emerging strategy involves co-delivery systems using micelles and nanoparticles to simultaneously carry peptides and small-molecule therapeutics in order to achieve synergistic therapeutic effects. In parallel, combining peptides with immunotherapeutic agents also represents a promising approach for enhancing immune-mediated recognition and elimination of cancer cells. Several clinical studies have demonstrated the therapeutic potential of combining peptides with monoclonal antibody-based immunotherapies. Peptides are also being utilized as functional delivery components for other immunotherapeutic modalities, including mRNA vaccines [4,124].

Taken together, peptide-based therapeutic development is transitioning from a largely empirical process toward an integrated and intelligent framework in which artificial intelligence, molecular simulation, and experimental validation operate synergistically to enable the rational design of highly precise therapeutic agents. Such advances may ultimately facilitate the development of clinically viable peptide therapeutics and establish a broadly generalizable strategy for targeting historically undruggable protein–protein interactions.

## 7. Conclusions

Peptides represent a promising strategy for targeting undruggable proteins due to their potential to achieve high affinity and selectivity. However, the translation from laboratory discovery to clinical application remains highly challenging, primarily because of limitations in cellular permeability, bioavailability, and the associated development costs. To address these challenges, the integration of artificial intelligence has become increasingly important. The combination of AI-based design, structure prediction, and property prediction not only expands the search space for novel candidates but also enables the prioritization of the most promising peptides for experimental validation. In this context, the integration of computational approaches with advanced experimental screening platforms provides an efficient framework to accelerate discovery while reducing time and cost. Together, these strategies are expected to facilitate the identification of more effective peptide-based therapeutic.

## Figures and Tables

**Table 1:** Summary of peptides targeting traditionally undruggable cancer targets.

Peptide	Target	Mechanism of Action	Binding Affinity	Cellular Activity	Structural Modification	Ref.
SAH-SOS1A	KRAS	Mimics SOS1 *α*-helix to disrupt SOS1 binding	EC_50_ = 100–175 nM	IC_50_ = 5–15 μM	Hydrocarbon staple (*i*, *i* + 4)	[24]
Stapled Peptide 3	KRAS^G12C^	Mimics SOS1 *α*-helix to disrupt SOS1 binding	K_d_ = 7.67 μM	IC_50_ = 15.69 μM; induces G2/M arrest and apoptosis	Truncated 11-mer; hydrocarbon staple (*i*, *i* + 4)	[25]
KRpep-2d	KRAS^G12D^	Binds Switch II/*α*3 helix; blocks SOS1 activation	IC_50_ = 1.6 nM; K_d_ = 8.9 nM	pERK inhibition at 30 μM; poor permeability	Cyclic (disulfide bond); 8 Arginine tails	[27,28]
KS-58	KRAS^G12D^	Binds allosteric Switch II site; blocks Ras-SOS1 and Ras-BRAF	EC_50_ ≈ 22 nM	IC_50_ ≈ 30 μM; inhibits pERK; in vivo tumor suppression	Bicyclic (amide + thioether); unnatural amino acids	[23]
Peptide 7	KRAS^G12D^	Dual inhibition: blocks SOS1 activation and Raf interaction	Subnanomolar (IC_50_ < 1 nM)	IC_50_ = 3.8 μM (pERK); improved permeability	Cyclic; thioacetal bridge; *α*-Me-Ser10;	[30]
Sraf-7-1	KRAS^G12C^	Blocks RAS-RAF interaction; disrupts PI3K pathway	K_d_ = 2.62 μM	IC_50_ = 8.84 μM; inhibits migration and induces apoptosis	Stapled helix; N-terminal stearic acid tag for permeability	[26]
Cyclorasin B4-27	Pan-Ras (Active state)	Blocks Ras interaction with downstream effectors (e.g., Raf)	K_d_ = 21 nM (for G12V)	EC_50_ = 2.1 μM; robust in vivo antitumor activity	Bicyclic; unnatural amino acids	[5]
LUNA18	Pan-KRAS (G12D/V/C)	Blocks KRAS-SOS interaction via induced-fit (SII-hole)	K_d_ = 0.043 nM	IC_50_ = 1.4 nM; Orally bioavailable (21–47%)	Cyclic; dense *N*-alkylation; rigid backbone	[6]
SAH-p53-8	p53-MDM2/MDMX interaction	Dual MDM2/MDMX inhibition, restoring p53 activity	K_d_ = 55 nM (MDM2)	EC_50_ = 8.8 μM (serum-free); inactive in standard 10% serum	Stapled peptide	[32,33]
ATSP-7041	p53-MDM2/MDMX interaction	Dual MDM2/MDMX inhibition, restoring p53 activity	K_d_ = 0.91 nM (MDM2), 2.31 nM (MDMX)	EC_50_ = 0.6 μM (SJSA-1 cells in 10% serum)	Stapled peptide (*i*, *i* + 7); utilizes cyclobutylalanine (Cba)	[34]
ALRN-6924 (Sulanemadlin)	p53-MDM2/MDMX interaction	Dual MDM2/MDMX inhibition, restoring p53 activity	K_d_ = 10.9 nM (MDM2), 57 nM (MDMX)	EC_50_ = 0.3 µM	Stapled α-helix (*i*, *i* + 7 hydrocarbon staple); polyalanine C-tail.	[33]
p53-16	p53-MDM2/MDMX interaction	Dual MDM2/MDMX inhibition, restoring p53 activity	K_d_ = 260 nM (MDM2), 180 nM (MDMX)	EC_50_ = 17.08 μM (MCF-7 cell viability)	Bicyclic (hydrocarbon + lactam staples)	[35]
MP-467	p53-MDM2/MDMX interaction	Dual MDM2/MDMX inhibition, restoring p53 activity	K_d_ = 25.54 nM	EC_50_ = 13 nM (HCT116 cell proliferation)	Di-alkyne rigid staple (*i*, *i* + 7); optimized polyAla tail.	[36]
PS10	p53-MDM2/MDMX interaction	Dual MDM2/MDMX inhibition, restoring p53 activity	K_d_ = 26 nM (MDM2)	Suppresses proliferation at 200 μM	Sulfono-γ-AApeptide scaffold; stapled peptide	[37]
DPMI-ω	p53-MDM2/MDMX interaction	Dual MDM2/MDMX inhibition, restoring p53 activity	K_d_ = 0.16 nM (MDM2), 28.7 nM (MDMX)	EC_50_ = 134.6 nM (delivered via gold nanoparticles)	Mirror-image D-peptide; modified Phenylalanine.	[38]
SPMI-HIF2-1	MDM2/MDMX (degradation)	SP-PROTAC; recruits VHL E3 ligase to trigger proteasomal destruction	K_i_ = 35 nM (MDM2), 53 nM (MDMX)	EC_50_ = 6.7 μM (HCT116 cell viability)	Stapled peptide (*i*, *i* + 7); Peg linker; LA-Hyp-Y-Hle-P ligand	[39]
ReACp53	Mutant p53 (aggregates)	Aggregation inhibitor	Targets adhesive region 251–258 of the p53 core domain	EC_50_ values in the low micromolar range	N-terminal poly-Arg tag	[40]
pCAPs (e.g., pCAP-250)	Mutant p53 (misfolded)	Conformation-activating peptide; stabilizes the rare active WT-like fold	K_d_ ~ 21 μM (binding to properly folded p53 DBD)	High efficacy in cell viability screens	Derived from phage display; N-terminal myristic acid	[41]
OMO-103 (Omomyc)	MYC	Sequestrates MYC into inactive dimers, blocks E-box binding, promotes MYC degradation	Nanomolar range for E-box binding	Shutdown of MYC gene signatures, stable disease in human patients, reduced tumor volume	91-aa miniprotein, 4 amino acid substitutions in the leucine zipper	[42]
IDP-121	c-MYC	Disrupts MYC-MAX dimerization; induces proteasomal degradation of unstable c-MYC monomers	K_d_ = 400 nM (10× higher than native interaction)	reduced viability of a large set of MM cell lines; induces apoptosis	Stapled peptide	[43,44]
NT-B2R	c-MYC	Transcriptional inhibitor; suppresses MYC-driven signatures by binding to a specific epitope	EC_50_ = 1680 nM	Suppressed MYC transcription activities and cell proliferation	Stereodiversified bicyclic peptide; synthesized via ROM-RCM; endo-R isomer	[45]
ME47 (MAX-E47)	DNA E-box (MYC binding site)	Competitive inhibition; occupies E-box DNA sites to physically block c-MYC/MAX access	K_d_ = 15.3 nM for E-box	Decreased DNA synthesis; negatively impacts the growth of MDA-MB-231 breast cancer cells	66-aa Minimalist Hybrid Protein (MHP); MAX basic region + E47 HLH domain	[46]
Max * (Max bHLHZip)	DNA E-box/c-MYC network	E-box competition; reactivates tumor suppressor genes typically repressed by c-MYC (lacks Miz-1 interaction)	“High affinity” competitive binding	Reduces metabolism and proliferation; G1 cell cycle arrest; induces apoptosis	83-aa mini-protein; contains an intrinsic Protein Transduction Domain (PTD)	[47]

**Table 2:** Summary of AI-driven tools for de novo peptide design.

Classification	Tool Name	Architecture	Subjects	Performance Metrics	Required Inputs	Source
Generative Design	RFdiffusion	De novo structural generation using a Denoising Diffusion Probabilistic Model (DDPM) fine-tuned on RoseTTAFold, utilizing frame representation and self-conditioning.	De novo design (Monomers, Binders, Scaffolds, Functional motifs, and symmetric Oligomers).	High experimental success with sub-angstrom accuracy (RMSD < 1 Å) and picomolar-affinity binding.	Target structure (PDB), motif/scaffold specifications, or symmetry constraints.	[8,85]
	RoseTTAFold Inpainting (RFjoint)	RoseTTAFold Inpainting network designed for simultaneous sequence and structural recovery (inpainting) of missing protein segments.	Functional site scaffolding and protein inpainting.	Demonstrated success in scaffolding functional sites with nanomolar target affinity.	Target structure and partial scaffold/binder coordinates for inpainting.	[8]
	BindCraft	Scaffold-free binder design pipeline using AlphaFold2 hallucination to optimize peptide-target interfaces directly.	Target-specific binders (Peptides and miniproteins).	Consistent generation of high-affinity binders with nanomolar potency (K_d_ in 65–650 nM range).	Target protein structure (PDB), target hotspots, and desired peptide length.	[77]
	Rosetta	Physics-based energy minimization and combinatorial sequence design using the PackRotamersMover algorithm.	Protein design and small molecule binding pocket optimization.	Robust physically based sequence recovery (~33%) and energy minimization.	Protein backbone structure (PDB) and physics-based energy functions.	[72]
Sequence Optimization	ProteinMPNN	Message-passing neural network (MPNN) with 3 encoder/decoder layers, random decoding order, and edge updates.	Sequence design (Monomers, Cyclic Oligomers, Nanoparticles, and Protein interfaces).	High sequence recovery (>50%) and structural stability for both monomers and nanoparticles.	Backbone coordinates (N, C_α_, C, O) and optional bias/fixed residue lists.	[8,72,77,85]
	AMPGen	Autoregressive diffusion generator guided by Evolutionary information (MSA) and integrated with XGBoost for bioactivity filtering.	Antimicrobial peptides (AMPs) (Sequence generation and screening).	Accurate prediction of bioactivity via Minimum Inhibitory Concentration (MIC).	Amino acid sequences and Evolutionary information (MSA) features.	[77]
	VAE-MH process	Generative modeling using a GRU-based Variational Autoencoder (VAE) coupled with Metropolis–Hastings (MH) sampling for latent space exploration.	Inhibitory peptides (Targeting *β*-catenin and NEMO pathways).	Effective discovery of potent inhibitors with low-micromolar activity (μM range).	Seed amino acid sequences and latent space parameters.	[77,100]
Structure Prediction and Validation	AlphaFold 3	Diffusion module and Pairformer blocks (replacing Evoformer); directly predicts raw atom coordinates.	Biomolecular complexes (Proteins, Nucleic acids, Small molecules/Ligands, Ions, and Glycans).	High atomic accuracy (RMSD < 1 Å for backbones) and superior ligand docking (>75% success rate).	Amino acid sequences, Nucleic acid sequences, SMILES (Ligands), and modification definitions.	[77,85,95]
	RoseTTAFold All-Atom (RFAA)	Generalized biomolecular modeling and design framework using a Frame-Aligned Point Error (FAPE) loss during prediction.	All-atom modeling (Proteins, Nucleic acids, Ligands, and covalent modifications).	Competitive ligand docking performance (>40% success rate in PoseBusters benchmark).	Amino acid sequences and chemical structures (SMILES) for ligands.	[77,85,95,101]
	ColabFold	AlphaFold2-powered tool for accessible and fast protein folding/multimer prediction.	High-throughput protein and multimer structure prediction.	Accelerated structure prediction with accuracy comparable to AlphaFold2.	Amino acid sequences (uses fast MMseqs2 for MSA generation).	[102]
	AlphaFold	End-to-end protein structure prediction using a Transformer-based architecture with Evoformer blocks and a structural module.	Protein folding (Structure prediction and de novo hallucination).	Industry standard for folding with high confidence scores (pLDDT > 80).	Amino acid sequences and Multiple Sequence Alignments (MSA).	[72]
	RoseTTAFold	Three-track neural network.	Proteins and Protein–protein complexes.	High accuracy in modeling complex protein–protein interactions (TM-score/RMSD).	Amino acid sequences and MSA features.	[72]
	PepNN	Graph Neural Network (GNN) using multi-head reciprocal attention layers to model peptide–protein interactions.	Peptide–protein interactions (Binding site and structure prediction).	High precision in mapping peptide–protein interfaces and binding sites.	Protein/Amino acid sequences and structural coordinates (PDB).	[77]
Specialized Scaffolds	AfCycDesign	AlphaFold2-based sequence-structure validation with modified positional encoding matrices tailored for cyclic peptide topologies.	Cyclic peptides (Structure prediction and sequence design).	Reliable cyclic peptide modeling with high structural fidelity (RMSD < 1 Å).	Amino acid sequences and desired cyclic topology constraints.	[77,101,103]
	HighFold	AlphaFold2-derived model incorporating specific constraints for disulfide bond pairing and cyclic backbone geometry.	Constrained peptides (Cyclic and Bicyclic scaffolds).	Enhanced structural accuracy for constrained peptides compared to standard AlphaFold2.	Amino acid sequences and specific disulfide/cyclic constraints.	[101]
	EvoPepFold	Hybrid framework combining Genetic Algorithms (GA) for sequence evolution with Rosetta docking and ColabFold for 3D structural fitness evaluation.	Peptide inhibitors (Protein–peptide docking and design).	Optimized binding energy (ΔG approx. −24 kcal/mol) and improved Rosetta energy scores.	Target protein structure and initial amino acid sequence library (e.g., from Propedia).	[102]
	streaMLine	High-throughput QSAR analysis pipeline utilizing Random Forest models to screen and optimize large-scale systematic peptide libraries.	Therapeutic peptides (e.g., GLP-1R agonists optimization).	Validated via pEC_50_ and solubility metrics for large-scale library screening.	Amino acid sequences (encoded via z-scales/one-hot) and assay measurement data.	[104]

**Table 3:** Opportunities and Limitations of AI in Peptide Design for Challenging Cancer Targets.

Target Class	Representative Target	Challenge	Representative Peptide Modalities	Clinical Maturity	AI-Design	
					Opportunity	Limitation
Small GTPases	KRAS	Lack of well-defined binding pockets High affinity for GTP, making competitive inhibition difficult	Cyclic peptides N-methylated peptides	Phase I	Allosteric & Transient Targeting: Employs generative models to reconstruct peptide scaffolds, enabling the coverage of large, flat oncogenic surfaces and the identification of transient cryptic pockets missed by traditional methods.	Transient Pocket Dependency: While AI can uncover cryptic pockets, their temporary and dynamic nature makes it highly complex for generative models to design scaffolds that maintain stable, high-affinity binding over time.
Protein–protein interaction targets	p53/MDM2	Large and relatively flat protein–protein interaction interfaces	Stapled peptide Cyclic peptides	Phase I-II	Conformational Complementarity: Optimizes geometric fit by expanding the buried surface area and precisely orienting hydrophobic anchors, driving binding affinity to picomolar levels.	Shallow & Hydrophobic Cleft: AI must balance engineering stable hydrophobic anchors (Phe19, Trp23, Leu26) with preventing excessive hydrophobicity that compromises the peptide’s aqueous solubility.
Intrinsically disordered proteins	c-MYC	Intrinsically disordered protein (IDP) lacking a stable three-dimensional structure	Mini-proteins Stapled peptides	Phase I-II	Dynamic Ensemble Stabilization: Utilizes structural modeling to stabilize specific target segments (e.g., LC46), facilitating the transition from disordered states to functional folded conformations to effectively block dimerization.	Static Model Failure on IDPs: Generative algorithms rely on static templates, failing to map the fluid dynamic conformational ensembles of IDPs. Spurious Structural Order: AI tools exhibit a bias toward imposing artificial, overly compact structural order onto naturally disordered regions.

## Data Availability

No new data were created or analyzed in this study. Data sharing is not applicable to this article.

## Abbreviations

3D	Three-Dimensional
ALIS	Automated Ligand Identification System
bHLHZip	basic Helix–Loop–Helix leucine Zipper
DNA	Deoxyribonucleic Acid
E-box	Enhancer box
GANs	Generative Adversarial Networks
GDP	Guanosine Diphosphate
GTP	Guanosine Triphosphate
MD	Molecular Dynamics
MSA	Multiple Sequence Alignment
PPIs	Protein–Protein Interactions
RNA	Ribonucleic Acid
SDEs	Stochastic Differential Equations
SII	Switch II
TAD	Transactivation Domain
VAEs	Variational Autoencoders
